# DNA methylation dynamics during stress response in woodland strawberry (*Fragaria vesca*)

**DOI:** 10.1093/hr/uhac174

**Published:** 2022-08-04

**Authors:** María-Estefanía López, David Roquis, Claude Becker, Béatrice Denoyes, Etienne Bucher

**Affiliations:** Crop Genome Dynamics Group, Agroscope, 1260 Nyon, Switzerland; Department of Botany and Plant Biology, Faculty of Sciences, University of Geneva, 1205 Geneva, Switzerland; Crop Genome Dynamics Group, Agroscope, 1260 Nyon, Switzerland; LMU BioCenter, Faculty of Biology, Ludwig-Maximilians-University Munich, D-82152 Martinsried, Germany; Univ. Bordeaux, INRAE, Biologie du Fruit et Pathologie, F-33140 Villenave d’Ornon, France; Crop Genome Dynamics Group, Agroscope, 1260 Nyon, Switzerland

## Abstract

Environmental stresses can result in a wide range of physiological and molecular responses in plants. These responses can also impact epigenetic information in genomes, especially at the level of DNA methylation (5-methylcytosine). DNA methylation is the hallmark heritable epigenetic modification and plays a key role in silencing transposable elements (TEs). Although DNA methylation is an essential epigenetic mechanism, fundamental aspects of its contribution to stress responses and adaptation remain obscure.

We investigated epigenome dynamics of wild strawberry (*Fragaria vesca*) in response to variable ecologically relevant environmental conditions at the DNA methylation level. *F. vesca* methylome responded with great plasticity to ecologically relevant abiotic and hormonal stresses. Thermal stress resulted in substantial genome-wide loss of DNA methylation. Notably, all tested stress conditions resulted in marked hot spots of differential DNA methylation near centromeric or pericentromeric regions, particularly in the non-symmetrical DNA methylation context. Additionally, we identified differentially methylated regions (DMRs) within promoter regions of transcription factor (TF) superfamilies involved in plant stress-response and assessed the effects of these changes on gene expression.

These findings improve our understanding on stress-response at the epigenome level by highlighting the correlation between DNA methylation, TEs and gene expression regulation in plants subjected to a broad range of environmental stresses.

## Introduction

Plants in natural environments are exposed to multiple stimuli, including numerous biotic and abiotic stresses that make it necessary for plants to develop strategies to rapidly adapt. According to the Global Climate Report 2020, the past 10 years were the warmest recorded around the globe in our era. The greater temperature variability has resulted in both droughts and extreme precipitations, affecting not only natural plant populations but also crop production [1]. In order to face these challenges, we need to better understand the mechanisms which allow plants to rapidly adapt and evolve to better cope with increasing climate change-related stresses. Recent advances in genome sequencing have revealed how dynamic plant genomes can be under stressful scenarios [2,3]. This dynamism can be attributed to both genetic and epigenetic mechanisms which can contribute to specific traits [4,5]. However, how epigenetic information is influenced by stresses [5,6] and whether these can contribute to adaptation requires a better understanding. One key epigenetic mark is DNA methylation (5-methylcytosine) which exists in three sequence contexts in plants: CG, CHG, and CHH (H = A, C, or T). Each of them is regulated by distinct, but also interconnected silencing mechanisms [7]. Symmetric methylation in the CG sequence context (mCG) has been found to be enriched in gene bodies but the biological function of gene body methylation (gbM) remains unclear [8]. mCG is highly heritable and able to persist over many generations. Conversely, DNA methylation in the CHG (mCHG) and CHH (mCHH) sequence contexts show a lower stability [9]. DNA methylation is a hallmark epigenetic modification, contributing to the regulation of many biological processes such as genome stability, definition of euchromatin and heterochromatin, control of gene expression, and, most importantly, silencing of transposable elements (TEs) [3,7]. Studies of DNA methylation variability in natural *Arabidopsis* accessions have shown a clear correlation between epigenomic changes in coding and non-coding genomic regions and environmental stimuli, suggesting a role for DNA methylation in adaptation [10]. More generally, it has been found that DNA methylation changes may be implicated in morphological changes in response to different climates in plants [11]. It has been suggested that DNA methylation might play a role in stress tolerance acquisition in plants. For instance, chilling stress in tea plant (*Camellia sinensis*) was found to cause hypomethylation in cold-responsive genes greatly enhancing their transcription thereby contributing to low temperature tolerance [12]. Similarly, low DNA methylation over gene bodies in seagrasses (*Posidonia oceanica* and *Cymodocea nodosa*) when sea-surface temperature increased showed transcriptional flexibility as an adaptive mechanism under changing environments [13]. Extreme water deficiency can result in DNA hypomethylation and upregulation of genes in the resurrection plant *Boea hygrometric* suggesting a rapid regulation of the stress-response machinery to contribute to dehydration tolerance [14]. In rice, it was show that a high affinity potassium transporter gene is regulated by DNA methylation in its promoter in order to maintain potassium and sodium homeostasis during salinity stress [15]. A notable example concerning environmental impacts on DNA methylation is the documented loss of methylation at TEs at high altitudes and how it may contribute to local adaptation in natural populations of wild strawberry [16,17]. However, whether stress-induced methylome alterations at individual loci or across the entire genome contribute to heritable changes in DNA methylation patterns and adaptation remains uncertain.

In the case of (octoploid) strawberry, it has previously been well described how abiotic stresses influence the normal development of the plant. To illustrate, in strawberry cultivars, drought stress was shown to affect chlorophyll, proline, and soluble carbohydrates levels. After rewatering, only proline and soluble carbohydrates went back to initial levels [18]. Low and high temperatures are known to disturb strawberry yield and fruit quality. ﻿For example, at high temperatures, strawberries initiate the production of chemical compounds which stunt plant growth [19]. Similarly, cold negatively affected anthocyanin content and the accumulation of soluble sugars in strawberry fruits [20]. Furthermore, hormonal levels (indole-acetic acid and abscisic acid) in seeds were also affected by low temperatures [20]. Higher tolerance of salt stress in strawberry cultivars have been associated with low stomatal density. However, salt stress caused accumulation of toxic ions in the leaves of non-salt tolerant strawberry plants [21,22]. Plants in low light had low photosynthetic rate with an increase of photosynthetic pigments, reduced stomatal conductance and altered sugar accumulation [23]. Conversely, at high light conditions, leaf height and photosynthetic rates augmented and as consequence, affected plant growth [24]. Finally, it was previously shown that SA increased vegetative growth and early flowering [22,25]. However, little was so far known about how these stresses can influence the epigenome of strawberry.

Here, we wanted to assess how a broad spectrum of well-established, ecologically relevant and climate change-related stresses can influence the epigenome of *F. vesca* (diploid strawberry). From an epigenetic point of view, *F. vesca* is an interesting model plant as it can multiply clonally (via stolons) or sexually (via seeds) [26]. Furthermore, in this species DNA methylation regulates key developmental traits such as seed dormancy [27] and fruit ripening [26]. Overall, we identified genomic regions which may act as stress-responsive epigenetic rheostats as a strategy to maintain homeostasis during unfavorable growth conditions in *F. vesca*. Our findings can be used to target epigenetic regulated genes for improvement of adaptation or stress tolerance in crops through epigenome editing.

## Results

### Stress-induced DNA methylation dynamics in *F. vesca*

**Figure 1:**
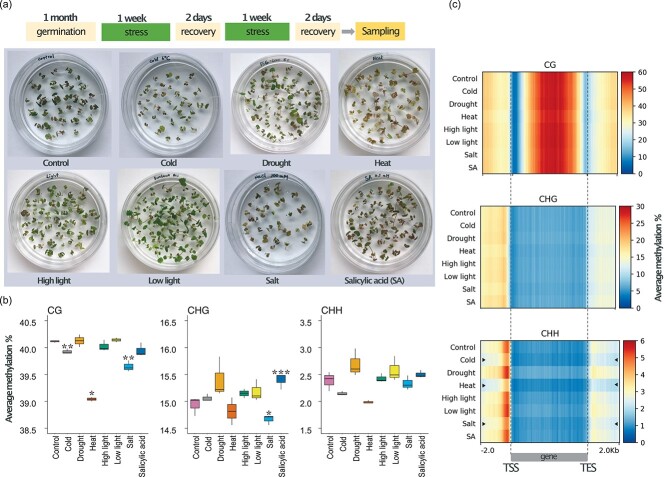
Effect of abiotic and hormone stresses on genome-wide DNA methylation levels in *F. vesca*. (a) Top: Scheme of stress treatment time course. Bottom: Photographs of plates with one-month-old plants grown under different stress conditions (cold, drought, heat, high and low intensity of light, salt, and salicylic acid). (b) Average DNA methylation levels for each cytosine context (CG, CHG, CHH) between normal and stress conditions (only common cytosines positions among all samples were considered that had a minimum coverage of 5 reads). Asterisks indicate levels of significance between treated and control plants: ^*^, p-value <0.05; ^**^, p-value <0.01; ^***^, p-value <0.001(﻿unpaired two-tailed Student’s t-test). (c) Heat maps showing distribution of DNA methylation (top: CG, middle: CHG, and bottom: CHH context) around genes with and without stress (Control). Mean of the average methylation percentage (within a sliding 100-bp window) was plotted 2 kb upstream of TSS, over the gene body and 2 kb downstream of TES. Black arrows highlight the samples in which a reduction of DNA methylation can be observed in the vicinity of TSS and TES sites.

To explore the ecological relevance and the dynamism of DNA methylation following stress exposure, we focused on multiple stimuli which plants may encounter in natural environments (Fig. 1a, see Materials and Methods for details). Here, *F. vesca* plants were submitted to two successive stress applications *in vitro*. To assess potentially stable DNA methylation changes we sampled the plants two days after the second stress. Compared to controls, stressed plants showed: reduce size after cold- and salt stress; tissue necrosis following heat stress; petiole elongation under low light; and reddish tissue coloration following SA exposure suggesting the accumulation of anthocyanins (Fig. 1a, Fig. S3). To assess genome-wide DNA methylation levels, DNA was extracted from these plants and submitted to whole genome bisulfite sequencing (WGBS, 20x genome coverage) (Table S1). We carried out a global quantification of DNA methylation in the three sequence contexts (CG, CHG and CHH). Overall, the global DNA methylation levels in all stress conditions were similar (Fig. 1b). *F. vesca* seedlings in control conditions had 40.11% mCG, 14.93% mCHG and 2.38% mCHH. We found significant decreases of 0.5%, 3.2% and 1.1% for global mCG after cold stress, heat stress and salt stress, respectively (Fig. 1b). In addition, we observed a significant global mCHG decrease of 1.8% in salt and an increase of 3.1% in the presence of SA. We did not detect significant changes in global mCHH level. To assess methylation variation in genic and non-genic sequences, we screened the methylome data in all three sequence contexts separately in three regions: 2 kb upstream of genes, along the gene body, and 2 kb downstream of genes using 100-bp sliding windows (Fig. 1c). In contrast to the global analysis, here we observed a higher local DNA methylation variability in the CHH context at the transcription start and end sites (TSS and TES, correspondingly) compared to the other two contexts. Notably for the CHH context, cold stress, heat stress and salt stress resulted in hypomethylation at the TSS, TES and over the gene body (Fig. 1c). We extracted genes with body methylation (gbM) similarly to the parameters defined previously [8] filtering for at least 20 CGs and a methylation level above the median value. Genes containing gbM showed low variability in all the conditions (Fig. S4).

### Stress particularly affects DNA methylation variability in the non-CG contexts in *F. vesca*

**Figure 2:**
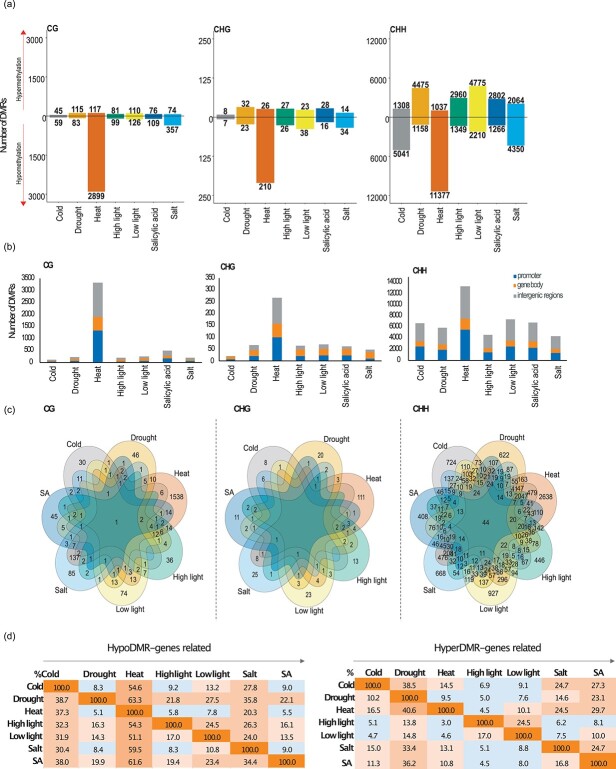
Differentially methylated regions in *F. vesca* seedlings grown at control and stress conditions. (a) Number of stress-induced hyper- (hyperDMRs) and hypomethylated DMRs (hypoDMRs) separated by sequence context. (b) Distribution of DMRs in genomic features: promoter (2 kb to 50 bp upstream TSS), gene body, and intergenic regions. Minimum overlap required: 1 bp. (c) Venn diagrams of common promoter and genic regions containing hypo- and hyperDMRs per context among all the stress conditions. (d) Percentages of genic loci with hypo- and hyperDMRs which are shared among the stress conditions. Reading from left to the right (arrow). e. g. (Left block) 8.3% genic locations with cold-stressed hypoDMRs overlap with 38.7% genic locations with drought-stress induced hypoDMRs. (Right block) 24% genic loci with hyperDMRs from salt-stressed seedlings overlap with 11% genic loci with hyperDMRs resulting from SA treatment. Ascending intensity of block colors correlate with overlap percentages.

To explore the dynamics of DNA methylation at specific loci in detail, we assessed differentially methylated regions (DMRs) for each sequence context. DMRs were defined using metilene v.0.2.6.1 which uses an algorithm to identify a base-pair window through sequence segmentation with significant methylation differences [32] (see Materials and Methods for parameters). We compared the methylomes of plants submitted to each stress condition with the methylomes from control plants. The majority of DMRs identified were in the CHH context for all conditions, with a maximum of 12 414 DMRs under heat stress. Fewer DMRs were detected in both CG sequence context, ranging from 104 (cold stress) to 3016 (heat stress), and in the CHG context, ranging from 15 (cold stress) to 236 (heat stress) (Fig. 2a, Table S2). In line with our global DNA methylation analysis, most of the heat stress, cold stress, and salt stress DMRs were hypomethylated (hypoDMRs) relative to the control condition in all sequence contexts. For drought stress, high light stress, low light stress, and SA stress, most of the DMRs showed hypermethylation (hyperDMRs) in the CHH context (Fig. 2a). Next, to test whether genic and non-genic regions were rich in DMRs, we qualified DMRs based on their intersection with promoters (empirically defined as 2 kb to 50 bp upstream TSS), gene bodies, and intergenic regions. The minimum overlap required was 1 bp. Many of the CG and CHG DMRs (30–44%, 45–60% respectively) were in genes, while most of the CHH DMRs (between 32–44%) were in promoters and intergenic regions (Fig. 2b). In summary, abiotic and hormone stresses led to DNA methylation changes primarily in the CHH context within promoters and intergenic regions. Overall, heat stress caused the most numerous DNA methylation changes.

### Identification of stress-induced DNA methylation changes in genic regions

**Figure 3:**
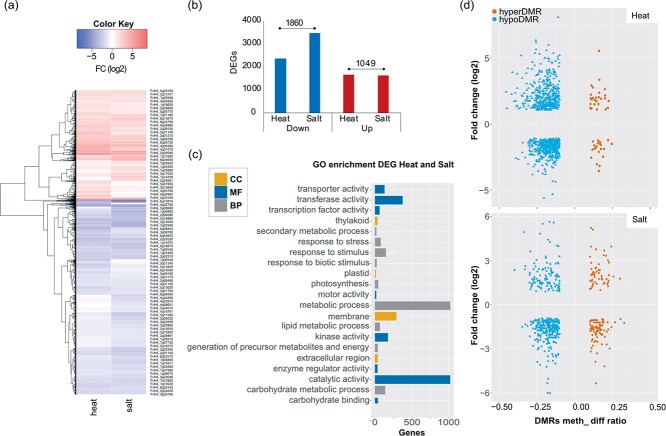
Functional analysis of differentially expressed genes (DEGs) after heat stress and salt stress. (a) Heatmap of DEGs of plants grown under heat stress and salt stress. Heatmaps were generated with log_2_ expression of counts normalized to transcript size and million mapped reads (DESeq2). DEGs are listed along the Y-axis in the order they clustered in as indicated by the colored line along the Y-axis. Each column contains expression values for individual genes, with groups indicated along the X-axis. Deeper red colors indicate higher expression while deeper blue indicates lower expression compared to the control. (b) Bar plots indicating the total number of DEGs in heat stress and salt stress. Intersected bars show the number of shared DEGs in heat stress and salt stress. (c) Singular enrichment analysis (SEA) of the total number of DEGs shared between heat stress and salt stress (results from AgriGOv2, p-adj:<0.05). The x-axis indicates the number of genes in a category. The y-axis indicates the most enriched GO terms in three categories: biological processes (BP, grey), cellular component (CC, orange), and molecular function (MF, dark blue). The x-axis indicates the number of genes in a category. (d) Scatter plot of DEGs related with DMRs (promoter and gene body) showing the relationship between transcript levels (fold change: log_2_) and DNA methylation (meth ratio). Top: heat stress and bottom: salt stress. Blue: hypoDMRs; Red: hyperDMRs.

To better understand the potential functional roles of the DMRs and the commonalities between the different stresses, we focused our analysis on DMRs located within promoters and gene bodies (Fig. 2c). We only identified one locus with a CG DMR (within *FvH4_6g40845* an unknown protein) and 44 loci with CHH DMRs that were in common to all stress conditions (Fig. 2c, Fig. S5). Comparing each stress data set of promoter and gene locations with hypo- and hyperDMRs we noted that heat stress shared more loci with hypoDMRs with the other conditions than all other comparisons (Fig. 2d, left). For example, 37.3% of loci with heat-stress hypoDMRs overlapped with 54.6% of all hypoDMRs found under cold-stress. Conversely, drought stress resulted in the highest number of genic loci with hyperDMRs shared with the other conditions. To illustrate, 33.4% of salt stress hyperDMRs overlapped with 14.6% drought stress hyperDMRs (Fig. 2d, right). In order to identify potential functional roles for these DMRs, we performed a singular enrichment analysis (SEA) using the AgriGO tool [38] (see Materials and Methods). *F. vesca* has around 34 000 genes but only 54% of which have been assigned a GO number [35]. For this reason, “unknown” annotated genes were omitted for this analysis (Table S3). The analysis was based on the identified genic regions (gene and promoter) with DMRs for each stress condition according to their methylation change (hypo- or hypermethylated). Plants submitted to heat stress showed thelargest variation in DNA methylation over genic regions. These were enriched with hyperDMR-associated genes involved in the generation of precursors of metabolites and energy. Genes with heat-stress induced hypoDMRs were enriched for transcription factor (TF) activity, transcriptional regulators, and genes related to cellular components (Table 1). We also found that cold stressed plants had hyperDMR-associated genes enriched for transporter activity (Table 1). Next, we wanted to correlate DNA methylation changes with transcriptional changes. For this analysis, we focused on heat stress and salt stress because of the significant loss of genome-wide DNA methylation that we observed for these conditions (Fig. 1b). To evaluate transcriptional changes, we performed RNA-sequencing on polyadenylated RNA extracted from the plants growing in control, heat stress, and salt stress conditions. We found 3966 and 5033 significantly differentiallyexpressed genes (DEGs) in heat stress and salt stress, respectively (Table S4, Table S5). To identify the functions of the DEGs we performed an enrichment analysis using the aforementioned parameters in the AgriGO tool (Fig. S6a). Interestingly, for heat stress we found genes enriched in reproductive process, pollination, and protein modification process. On the other hand, salt stress resulted in a specific response in pyrophosphatase activity, transport, motor activity, homeostatic and hydrolase activity. Even though distinct stress-response genes were responding to the two stresses, heat stress and salt stress shared a substantial number of DEGs with similar expression patterns (Fig. 3a). To illustrate, 1860 down-regulated DEGs and 1049 up-regulated DEGs were in common for both conditions (Fig. 3b). This group of genes in heat stress and salt stress were related with molecular functions such as catalytic activity, transferase activity, nucleotide binding and transcription factors. Biological process genes were related with metabolic processes, and response to stress. Finally, cellular components included cell wall and membrane (Fig. 3c). In order to identify possible pathways playing a role in heat stress and salt stress response we performed a KEGG enrichment analysis [39]. Genes related with enzymatic and transcription factor activity were enriched for both stresses. For example, peroxidases, reductases and MYB TFs following heat stress. Similarly, under salt stress, MYB TFs were the most enriched group of genes (Fig. S6b). Next, we wanted to identify if there was a relationship between the presence of DMRs in genic regions (gene and promoter) and transcriptional changes (Fig. 3d). We identified 172 heat stress DEGs and 124 salt stress DEGs with DMRs located in the gene body; 637 and 366 DEGs with promoter DMRs; 58 and 14 DEGs with DMRs in both gene body and promoter in each stress respectively. However, the presence of hypo- or hyperDMRs did not correlate with the transcription patterns (up- or down-regulation) of these genes (Fig. 3d). For instance, hypoDMRs were equally distributed between up- and down-regulated genes in both conditions. Additionally, we identified DEG with heat-stress DMRs which were enriched in transcription factor activity, reproductive process, membrane, and cell wall (Fig. S6c). In the case of salt stress, the enrichment representation of DEG with salt-stress DMRs showed genes involved in catalytic activity (Fig. S6c). Taken together, heat stress caused more DNA methylation changes while salt stress caused more transcriptional changes.

**Table 1:** Gene ontology (GO) enrichment analysis of genic regions (gene and promoter) with DMRs. GO enrichment of genes with hypo- and hyperDMRs caused by thermal stress. The genes are arranged according to their DMRs patterns

**Stress**	**Methylation**	**Description**	**FDR**	**Number in input list**	**Number in BG/Ref**	**Ontology**
Cold	Hypermethylation	transporter activity	0.024	23	838	F
Heat	Hypermethylation	generation of precursor metabolites and energy	0.021	8	153	P
	Hypomethylation	transcription regulator activity	0.024	108	420	F
		transcription factor activity	0.024	99	380	F
		intracellular	0.033	504	2276	C
		cell part	0.033	849	3930	C
		cell	0.033	849	3930	C
		cytoplasm	0.033	239	1029	C

GO categories are listed with the false discovery rate-adjusted P-value <0.05. BG: background, Ref: reference, C: Cellular Component; F: Molecular Function; P: Biological process. Only well identified and annotated genes were included in the analysis.

**Figure 4:**
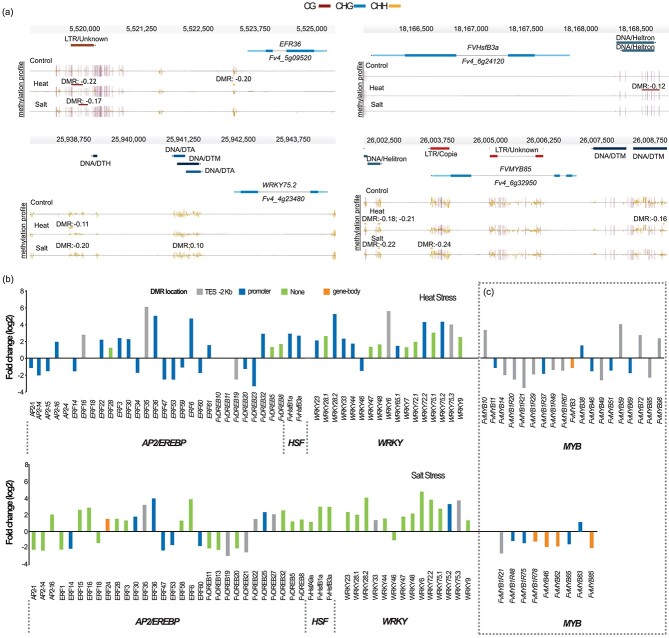
Stress induced hypoDMRs at transcription factor coding genes. Genome browser views of DMRs located in promoter regions of (a) APETALA2/ethylene-responsive element binding protein (AP2/EREBP) superfamily genes: ERF36 (ethylene-responsive binding factor 36); heat shock transcription factors (HSF) genes: FvHSFB3a (heat shock transcription factor B); WRKY superfamily: WRKY 75.2; MYB superfamily: FvMYB85. Depicted are genes structures (top panels, UTRs in light blue, exons in blue), TEs (red and dark blue) and DNA methylation levels (histograms). Boxes above the histograms indicate identified DMRs with methylation difference ratios (color codes for DNA methylation: red for CG, blue for CHG and yellow for CHH contexts). (b) Differentially expressed transcription factor (TF) families after heat stress. Transcription profiles of AP2/EREBP, HSF, WRKY, MYB genes. (c) selected differentially transcribed MYB genes related with a DMR (dashed box). Note that for space reasons all differentially expressed MYBs without DMRs have been omitted here. DMR location is indicated in the upper colored boxes: TES -2 kb (grey), promoter (blue), none (green). The x-axis indicates the TF member per family. The y-axis indicates fold change (log_2_) with p-adj. < 0.05.

### Stress induces hypomethylation and its effect on the expression of transcription factor coding genes

Transcription factors (TFs) play key roles in plant growth, development, and stress responses. Remarkably, we observed that heat stress resulted in an enrichment of hypoDMRs at promoters of genes related to TFs. Furthermore, heat-stress DEGs associated with DMRs also showed an enrichment in TFs (Fig. S6c). We found that under heat stress, 99 genes from the GO category transcription factor activity (Table 1) were associated with DMRs. Of these, 31% were *AP2/EREBP,* 19% *WRKY* genes and 4% heat shock transcription factors (*HSF*s). *AP2/EREBP* members (119 genes in total) have been characterized in the latest version of the *F. vesca* genome and are known to be involved in stress tolerance [40, 41]. To evaluate the detailed DNA methylation changes at *AP2/EREBP* genes under different stress conditions, we looked at the distribution of DNA methylation at those loci (Fig. S7a). We observed a noted reduction in DNA methylation at the TSS in the CHH context for heat-, cold- and salt-stressed plants. Combining all stress conditions, a total of 74 DMRs were identified within the promoter regions of 44 *AP2/EREBP* genes (Table S6). In addition, 14 *HSF*s have been identified in *F. vesca* genome [42]; however, in the last genome annotation version [35], we were able to identify 19 *HSFs* (Table S7). The distribution of methylation over *HSFs* showed high variability in TSS and TES in CHH context compared gene bodies (Fig. S7b). We identified 13 *HSFs* with DMRs within promoter regions mostly attributed to heat stress (Table S7). The marked presence of DMRs within TF promoters suggested a relationship between methylation and transcription. For this reason, we selected different members of TF families to evaluate changes in methylation and transcription (Fig. 4). Four examples with clear changes in DNA methylation in the promoters of the *AP2/EREBP*, *HSF*, *WRKY*, and *MYB* TF gene families are shown in Fig. 4a. We therefore extracted the list of members of the TF superfamilies to verify their transcript levels in our RNA-seq data for heat stress and salt stress (Fig. 4b). Overall, we identified 13 *AP2/EREBP* genes that were significantly up regulated in heat stress and 17 *AP2/EREBP* genes in salt stress (Fig. 4b). We also obtained similar results by analyzing the relationship between methylation and expression of heat shock factors (*HSFs*). Only *FvHSFB1a* and *FvHSFB3a* showed significant changes in transcription in heat stress (Fig. 4b). Hypomethylation in promoter regions of *FvHSFB1a* and *FvHSB3a* showed a clear relationship with significantly increased transcript levels after heat stress. However, in salt stress *FvHSFB1a* and *FvHSB3* were up regulated without being associated with a DMR. Similarly, in *F. vesca,* 61 *WRKY* and 217 *MYB* genes have been identified [35, 43, 44] and described to be differentially expressed under abiotic stress conditions. Here, as part of the DEGs analyzed, we found 16 *WRKY* genes to be up regulated by heat stress and 9 of which contained DMRs (Fig. 4b). On the contrary, 14 *WRKY* genes were differentially expressed after salt stress with almost no associated DMR. For the MYB superfamily, 48 genes were differentially expressed in heat stress and 64 in salt stress (Table S8). However, few of those were related with a DMR: 19 and 9 MYB TFs were related with a DMR in heat stress and salt stress respectively (Fig. 4c, Table S8). Collectively, our data show that heat stress and salt stress trigger the up- and down- regulation of TFs. Nonetheless, only heat-stress induces loss of DNA methylation mostly at promoter regions of genes related to specific TF families possibly influencing their expression. Overall, transcriptional changes seemed not to correlate with changes in DNA methylation.

### Stress leads to distinct methylation changes at transposable elements (TEs)

**Figure 5:**
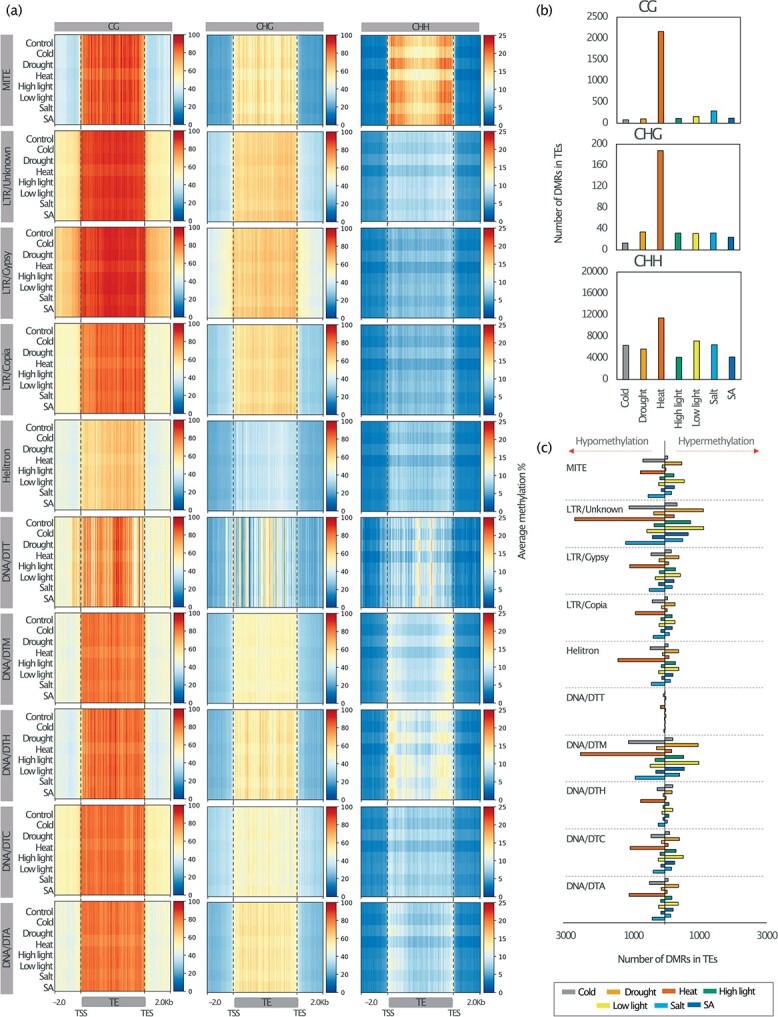
Association of stress-induced differentially methylated regions with transposable elements in *F. vesca*. (a) Heatmaps showing DNA methylation profiles for all the TE families separated by sequence context mCG (left), mCHG (center) and mCHH (right). The mean of the average DNA methylation percentage (within 50 bp sliding windows) was plotted for the TE bodies and 2 kb around the TSS and TES regions. (b) Number of stress induced DMRs in TEs per sequence context. (c) Number of hypoDMRs (left) and hyperDMRs (right) within different TE families. Class I elements (retrotransposons): LTR-Copia, LTR-Gypsy. Class II elements (DNA transposons): TIR: Tc1–Mariner (DTT), hAT (DTA), Mutator (DTM), PIF– Harbinger (DTH), CACTA (DTC); Helitron; Miniature Inverted-Repeat Transposons (MITEs). Upper char box indicates the colors which represent each treatment.

Since one of the most important functions of DNA methylation is to repress TE transcription and mobility [7,45], we investigated the effect of stress on DNA methylation at TEs. To describe the variation in DNA methylation in TE bodies and their flanking regions we plotted DNA methylation profiles in all three contexts. We used 50-bp sliding window: 2 kb upstream, over the body and 2 kb downstream (Fig. 5a). In general, all TE families showed high methylation levels in CG context; however, DNA transposons such as the *Mariner* (DTT) and *Helitrons* superfamilies were characterized by low DNA methylation levels in the CHG context. Notably, *Miniature Inverted-Repeat Transposons* (MITEs) showed the highest DNA methylation levels in CHH context. For MITEs, mCHH was distinctly reduced under cold, heat and salt stress (Fig. 5a). To assess significant changes in DNA methylation at specific loci over TEs, we counted the number of DMRs within TE annotations for each stress condition (Fig. 5b). The minimum overlap DMRs/TEs was 1 bp. As for genes, we identified the greatest number of DMRs within TEs in heat-stressed plants in all cytosine contexts (Fig. 5b). Cold stress, heat stress, and salt stress displayed more hypoDMRs in TEs compared to drought stress, low light stress, high light stress, and SA stress, which induced more hyperDMRs in TEs (Fig. 5c). Although there was no specific TE family significantly enriched in DMRs, heat stress resulted in at least 12% of all MITES acquiring hypoDMRs (Fig. 5c, Table S9) and most of them were close to genic regions (< 2 kb upstream genes) (see Fig. S8 for examples). Overall, these results suggest that DNA methylation dynamics among all TE members changed proportionally in all superfamilies; nonetheless, the change regarding gain or loss of DNA methylation was clearly defined by the applied stresses.

### DMRs are enriched in distinct regions of the *F. vesca* in the genome

**Figure 6:**
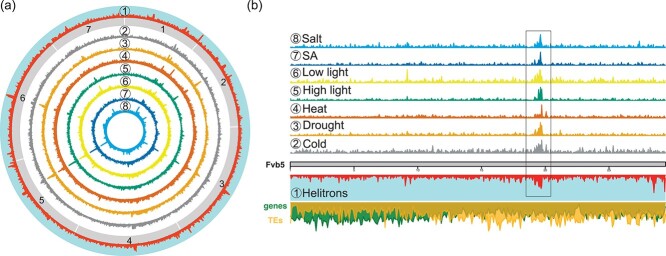
Genome-Wide distribution of DMR densities. (a) Circa plot showing DMR densities on all 7 strawberry chromosomes (gray boxes) for each stress condition (2. Cold, 3. Drought, 4. Heat, 5. High light, 6. Low light, 7. SA, and 8. Salt). *Helitron* density (1) in the genome is depicted on the outer most circle in red with turquoise background. (b) DMR density depicted on chromosome 5 (Fvb5) displaying a common enrichment among all the stress conditions (left of the 20 Mb tick mark). Below, the chromosome is indicated in grey, green shows gene density, yellow the TE density and red Helitrons along the chromosome.

Even though genome-wide DNA methylation variation levels were low (Fig. 1b), we observed regions in the genome that were enriched in DMRs in all stress conditions (Fig. 6a). Indeed, we observed DMR hotspots in the *F. vesca* genome. Notably, the distribution showed a similar pattern on all chromosomes independently of the stress conditions. The DMR density was not clearly related to gene and TE density (Fig. 6b); nevertheless, when we analyzed individual TE families, *Helitron* density hotspots showed a clear correlation with DMR density (Fig. 6). The *Helitron*-rich regions may highlight centromeric regions, however little is currently known about the exact localization of centromeric and pericentromeric regions in strawberry [46]. However, it has been reported previously that centromeric regions can be enriched in *Helitrons* in certain plant genomes [47]. It is important to note here, that the *Helitrons* themselves were not enriched in DMRs.

## Discussion

### Genome-wide DNA methylation patterns are altered under stressful environmental conditions

One genomic response of plants to abiotic and biotic stresses is to change their epigenome [5]. DNA methylation variation might trigger modifications in plant development and physiology contributing to phenotypic variability, thereby presumably contributing to plant acclimation [48]. Here, we wanted to investigate how these previously well-described stresses can affect DNA methylation at the genome-wide level. Looking at global DNA methylation levels in the three different sequence contexts, we observed only slight variations among the different stress conditions. However,when performing the DMR analyses we detected extensive differences at specific loci especially in the CHH context. This is in agreement with previous studies which reported that mCHH was most dynamic in response to different climates [9]. More specifically, in the case of *F. vesca*, altitude variations of natural strawberry populations was found to be correlated with a high variability in DNA methylation in the CHH context [16, 49]. In our study, cold stress, heat stress, and salt stress resulted in substantial local loss of DNA methylation in all sequence contexts, particularly in regions close to TSS and TES sites (Fig. 1c). Such DNA methylation changes have been associated with different degrees of cold tolerance in other plant species. For instance, chickpea presented an increased number of hypomethylated regions near abiotic stress response genes and transcription factors upon cold stress [50]. Similarly, higher temperatures resulted in hypomethylation in rice seeds, soybean roots, and tobacco leaves, affecting plant growth by altering gene expression patters in genes that control biosynthesis and catabolism of phytohormones [51–53]. In addition, salt stress-induced epigenetic variation in Arabidopsis has been shown to be partially transmitted to offspring, primarily via the female germ line [54]. On the other hand, we noticed some gain of global DNA methylation caused by different intensities of light or drought stress and SA treatments resulting a considerable number of hyperDMRs in the CHH context. Different results were found in tomato plants exposed to variable intensities of light which resulted in DNA hypomethylation and transcriptional changes causing male-sterility [55]. Furthermore, drought stress induced mCG and mCHG hypermethylation and a slight decrease in mCHH methylation in mulberry [56]. There is also evidence that drought, nitrogen-deficiency and heavy metal stresses can result in heritable changes in DNA methylation levels across generations in rice [57–59]. Another study showed that DNA methylation changes induced by hormone stresses via jasmonic and salicylic acid can be faithfully transmitted to offspring by asexual reproduction in dandelions [60]. Taken together, a common pattern in the responses of DNA methylation changes between plant species does not seem to exist. Our results raise an important question that remains to be tested: Are stress-induced DNA methylation changes in *F. vesca* maintained over generations through asexual (stolon) and sexual (seeds) reproduction? This is an intriguing aspect we are currently investigating.

### Different TE superfamilies show contrasted responses to stresses

DNA methylation plays a key role in limiting transcriptional activation and mobilization of TEs in order to ensure genome integrity [45, 61, 62]. Indeed, TEs can be an important source of genetic and epigenetic variation that can influence stress-responses [63]. Here, we wanted to better understand how different TE superfamilies respond to the stresses we applied. Using this approach, we observed distinct DNA methylation profiles at TEs depending on their superfamily and the applied stress conditions. Overall, we found that *F. vesca* TEs are highly methylated in both, CG and CHG sequence contexts. Similar results were observed in maize where LTR and TIR elements are highly methylated under normal conditions [64]. On the other hand, we noticed that overall DNA methylation levels in the CHH context were lower and more variable among different TE superfamilies and stress conditions, implying contrasted responses of TEs to stresses and that these TEs are silenced by different transcriptional gene silencing pathways. These DNA methylation changes could have direct physiological impacts as non-CG methylation seems to create a boundary between genes and TEs [9]. For example, it has been found that TEs located close to stress-induced genes in Arabidopsis and rice are silenced by hypermethylation after phosphate starvation in order to prevent collateral activation of TE transcription during stress [65]. In our study we found that heat stress affected mCHH in the TE body of all TE superfamilies. MITES had the highest percentage of members targeted by DMRs (Table S8 and Fig. S8). Previous studies have highlighted the importance of MITES in genome evolution and how MITE insertions in promoter regions can regulate the expression of genes in a wide variety plant species such as mulberry [66]. Taken together, these observations suggest that specific TE superfamily members with dynamic DNA methylation levels may contribute to stress response strategies in plants.

### DMR location preferences for centromeric regions

It has long been established that DNA methylation is enriched in peri−/centromeric regions which follow the distribution of TEs over the chromosomes of genomes of the Brassicaceae family, as recently also confirmed for *Thlaspi arvense* [67, 68]. In our study, while investigating the global distribution of DMRs over the *F. vesca* chromosomes, we found that regions with high DMR density correlated with regions enriched in *Helitrons*. Interestingly, we made this observation for all stress conditions (Fig. 6). Currently, *F. vesca* centromeres are not well defined; however, a genome-wide scan of the *F. vesca* genome for tandem repeats suggested the presence of *Helitrons* near centromeres [47]. This supports our hypothesis that *F. vesca* centromeres or pericentromeric regions respond to stresses with DNA methylation changes. To further confirm the exact localization of *F. vesca* centromeres, immunoprecipitations using a CENH3 antibody followed by sequencing will be required [69]. Why centromere-associated regions are more prone to DNA methylation changes and the physiological relevance of this observation still needs to be determined.

### Heat stress and salt stress trigger similar transcriptional responses but different magnitudes of DNA methylation changes

In our study, the global analysis of the DMRs resulting from each tested stress condition highlighted numerous shared genomic patterns among the stresses but also interesting stress-specific genomic features. In the case of heat stress, we observed hypoDMRs predominantly in gene, promoter, and TE regions. Extreme temperatures are one of the main stresses affecting plants that particularly alter their development and potentially cause yield loss [70]. The functional analysis of DEGs after heat stress in *F. vesca* showed an enrichment in genes related to stress response, reproduction process, photosynthesis, and metabolic process (Fig. S6a) which is similar to what has been previously described as an effect of the high temperatures [70]. Similarly, DEGs resulting from salt stress were related to external stimuli response, transporter activity, transcription factors, catabolic and metabolic processes (Fig. S6a). It has previously been described that heat stress and salt stress trigger the activation of common genes related with osmotic stress, oxidative stress and leaf physiology, and particularly, photosynthetic activity in plants such as olive, rice, and coffee [71, 72]. In this study we found that DMRs were enriched in genes related to transcription factor regulation and activity as well as generators of metabolites and energy for heat stress. This result is consistent with the idea that transcription factors are required to reprogram stress-related genes [73]. There is evidence demonstrating that heat stress responses can also have an effect on epigenetic regulators and small RNAs to rapidly activate genes [74]. However, little is known about how these responses are directly influenced by changes in DNA methylation or *vice versa*. Transcription factor families such as *AP2/EREBP*,*WRKY*, *MYB*, *HSFs* play important roles in response to abiotic stresses in plants [41, 44, 75]. To illustrate, in *Populus thricocarpa,* 1156 TFs (including MYB, AP2, WRKY, NAC, and bHLH) showed loss of methylation and changes in expression after drought stress [76]. Moreover, some *AP2/EREBP* genes are known to be highly induced under heat stress conditions by HSFs through an interconnected stress regulatory network [77]. Here, we provide epigenetic evidence suggesting that members of the *AP2/EREBP* TF class might be regulated by changes in DNA methylation in *F. vesca* after heat stress. On the other hand, salt stress resulted in the activation of similar TFs without accompanying DMRs in the gene body or promoter regions. Among them, the promoter region of the ethylene response factor (*ERF)* genes showed loss of methylation only after heat stress but significant up-regulation in heat stress and salt stress. Recent studies have shown that ERFs enhance basal thermotolerance by regulating heat-responsive genes and interacting with HSF in Arabidopsis and tomato [78, 79]. Similarly, hypomethylation in the TSS of genes involved in the control of cell growth in tobacco and stress-tolerance genes in maize after heat stress exposure is consistent with the increase in their transcription levels [51, 80]. Correspondingly, we identified 58% of *HSFs* genes with hypoDMRs in their promoter regions after heat stress. *HSF*s are crucial for thermotolerance capacity and regulate the expression of several heat-stress response genes such as heat shock proteins (HSPs) [77]. Here, we showed up-regulation of class B *HSF*s in *F. vesca* after heat and salt stress. Comparable results were obtained in a transcriptome analysis of the octoploid strawberry where *HSF* expression was induced by a heat shock treatment [81]. Notably, we found 26% of all *WRKY* genes to be differentially expressed in heat stress which is similar what was described by Wei *et al.,* (2016) who found 8 *WRKY* genes to be up-regulated at 42°C in *F. vesca* by semi quantitative PCR [43]. At the same time, in soybean *WRKY* genes were highly induced under salt stress conditions [82]. ﻿Concerning MYB TFs, it has previously been reported that they can be regulated by ﻿gibberellic acid, abscisic acid, cold, and heat treatments in *F. vesca* [44]. Markedly, differentially expressed MYB TFs were affected in another way at the epigenetic level. While after heat stress 40% of those MYB TFs were associated with a DMR, that was only the case for 14% of them after salt stress (Table S8). Taken together, these findings provide insights into stress induced DNA methylation as being partially independent from the transcriptional regulation. It will be of great interest to investigate the direct role of DNA hypomethylation in promoter regions of genes in regulating or priming transcription following heat stress and salt stress in *F. vesca*.

## Conclusions

In summary, our data revealed how DNA methylation profiles at genes and transposable elements can vary in response to stresses in wild strawberry. In addition, we observed changes in DNA methylation and gene expression changes do not necessarily coincide following stress exposure. These results show how versatile and plastic the epigenome can be under unfavorable conditions. Furthermore, we provide insights into how specific chromosomal regions can vary at DNA methylation levels under stress conditions. These observations suggest that the epigenetic flexibility of centromeres may play an important role during plant stress response. These results obtained with *F. vesca* as a model plant will help to better understand the stress response of more complex genomes in the Rosacea family. Overall, this study with high-resolution methylome mapping of the *F. vesca* genome will contribute to a better comprehension of epigenetic responses under variable growth conditions. It remains to be tested if such epigenetic changes can be inherited during sexual or clonal propagation (which is common in *F. vesca*) and if such changes could contribute to adaptation to changing environments.

## Materials and methods

### Plant growth and material

All strawberry plants used in this study were a homozygous cultivated near-isogenic line (NIL), Fb2:39–47, *F. vesca cv. Reine des Vallées* (RV), possessing the “r” locus on chromosome 2 which causes this accession to propagate vegetatively through stolon development [28]. Seeds from a single founder plant were germinated in water over Whatman filter paper for two weeks and transferred to 50% Murashige & Skoog (MS) medium (Duschefa cat# M0222), 30% sucrose, and 2% phytagel (Sigma-Aldrich cat# P8169) and grown for 4 weeks prior to stress.

### Stress assays

One-month-old seedlings on agarose plates were exposed to different stresses under long-day conditions (16 h light 24°C/8 h dark 21°C) in plant growth chambers (Panasonic, phcbi: MLR-352/MLR-352H)*.* The seedling age was optimized to assure stress tolerance. For salt and drought stress, one-month-old plantlets were transferred to MS media supplemented with 100 mM sodium chloride (NaCl) (Sigma-Aldrich, cat# S9888) and 5% polyethylene glycol (PEG-6000) (−0.05 MPa) (Sigma-Aldrich, cat# P7181), respectively. For cold and heat stresses, plants were initially grown as described above. The plates were then transferred to either 6°C or 37°C chambers. High light was induced by 20 000 lx of illuminance (460 μmol s^−1^ m^−2^) and low light with 80% sunblock black net leading to 4000 lx of illuminance (92 μmol s^−1^ m^−2^). To simulate a hormone stress, MS medium was supplemented with 0.5 mM salicylic acid (SA) (Sigma-Aldrich, cat# 247588). All stress assays were carried out for 2 weeks with 2 recovery days after one week. We sampled the following arial parts of plants: meristem, 2 cotyledon leaves, the first true leaf and the first trifoliate leaf. For molecular analysis, we chose 5 plants to reduce the variability among individual plants and three biological replicates for statistical analysis. The requirements for methylome sequencing has been well defined and standardized in different previous studies [29,30]. The experiments were designed to meet the requirements to run the necessary bioinformatic pipelines [31,32]. The samples were harvested in 1.5 mL tubes between 9:00 a.m. and 11:00 a.m. and immediately flash-frozen in liquid nitrogen and stored at -80°C.

### Genome sequencing and assembly NIL Fb2

Genomic DNA from strawberry plants was extracted by a Hexadecyltrimethylammonium bromide (Cetrimonium bromide, CTAB) modified protocol [28] and purified with Agencourt AMPure XP beads (cat# A63880). Long-read sequencing was performed for genome assembly; Genomic DNA by Ligation (Oxford Nanopore, cat# SQK-LSK109) library was prepared as described by the manufacturer and sequenced on a MinION for 72 h (Oxford Nanopore).

### Reference genome polishing

Reads obtained from nanopore were filtered with Filtlong v0.2.1 (https://github.com/rrwick/Filtlong) using --min_mean_q 80 and --min_length 200. Cleaned reads were then aligned to the most recent version of the *F. vesca* genome v4.0.a1 [33], with the annotation of *F. vesca* genome v4.0.a2 downloaded from the Genome Database for Rosaceae (GDR) (https://www.rosaceae.org/species/fragaria_vesca/genome_v4.0.a2), using minimap2 v2.21 [34] with parameters -aLx map-ont --MD -Y. The generated BAM file was then sorted and indexed with samtools v1.11. We used mosdepth v0.3.1 to verify that coverage on chromosomic scaffolds was over 50 X. Sniffles v1.0.12a with parameters: -s 10 -r 1000 -q 20 --genotype -l 30 -d 1000 was used to detect structural variations larger than 30 bp. We observed that larger structural variants (SV) were most likely falsely identified due to misalignments in regions with gaps or Ns, therefore the VCF files obtained from Sniffles were sorted and filtered with BCFtools v1.14 to keep only SV with less than 200 kb, supported by 10 or more reads and with allelic frequencies above 0.8 to isolate homozygous changes. The complete filtering command used was “bcftools view -q 0.8 -Oz -i ‘(SVTYPE=“DUP“ || SVTYPE=“INS” || SVTYPE = “DEL” || SVTYPE = “TRA” || SVTYPE = “INV” || SVTYPE = “INVDUP“) && %FILTER=“PASS“ && FMT/DV>9 && SVLEN>29 && SVLEN<200000’”. From the VCF listing all the structural variants that we detected in our *F. vesca* accession, we generated a substituted genome version based on the reference *F. vesca* genome v.4.0.a2. The reference genome was first indexed with samtools faidx v1.11 and a sequence dictionary were generated with Picard CreateSequenceDictionary v2.25.6 (https://broadinstitute.github.io/picard). The VCF containing the SV produced from our Nanopore sequencing was also indexed with IndexFeatureFile v4.2.0.0 (https://gatk.broadinstitute.org/hc/en-us/articles/360037262651-IndexFeatureFile). FastaAlternateReferenceMaker v4.2.0.0 (https://gatk.broadinstitute.org/hc/en-us/articles/360037594571-FastaAlternateReferenceMaker) was then run with the reference genome and the VCF file to generate a substituted genome representative of our *Fragaria* accession (Fb2). As substituting our genome with the detected structural variants changes genomic coordinates, we also corrected the public GFF genome annotation of *F. vesca* [35] using liftoff v1.6.1. Liftoff also detects and annotates duplications within the substituted genome. Transposable elements annotation was carried out using the EDTA transposable element annotation pipeline v. 1.9.6 [36] on the substituted genome using default parameters*.*

### Whole-genome bisulfite sequencing (WGBS)

A modified CTAB DNA extraction protocol was performed using frozen above-ground tissues [28]. DNA libraries were generated using the NEBNext Ultra II DNA Library Prep Kit (New England Biolabs, cat# E7103S) according to the manufacturer’s instructions with the following modification for bisulfite treatment. DNA was sheared to 350 bp using a Covaris S2 instrument. The bisulfite treatment step using the EZ DNA Methylation-Gold kit (Zymo Research, cat# D5007) was inserted after the adaptor ligation. After clean-up of the bisulfite conversion reaction, library enrichment was done using Kapa Hifi Uracil+ DNA polymerase (Kapa Biosystems, cat# KK1512) for 12 PCR cycles, using the 96 single-index NEBNext Multiplex Oligos for Illumina (New England Biolabs, cat# E7335S). Paired-end reads were obtained on an Illumina (150 bps) NovaSeq6000 instrument at Novogene (Hongkong, China).

### Processing and alignment of bisulfite-converted reads

Sequencing data was analyzed by data collection software from read alignment to DNA methylation analysis: Epidiverse/wgbs pipeline [31]. The pipeline included quality control using FastQC v.0.11.9 (http://www.bioinformatics.babraham.ac.uk/projects/fastqc/) and Cutadapt v.3.5 (https://github.com/marcelm/cutadapt/). Genome mapping was performed using erne-bs5 v.2.1.1 with default parameters to generate the BAM files. Methylation calling and methylation bias correction was performed with Methyldackel v.0.6.1 (https://github.com/dpryan79/MethylDackel) with only uniquely-mapping reads. The pipeline used Nextflow v20.07.1 to run multitask in parallel. Because plant chloroplast DNA are not methylated [37], reads originating from those sequenced were used to evaluate the bisulfite conversion rate. The pipeline is available at https://github.com/EpiDiverse/wgbs. An average of 82 771 701 reads (~ 50X coverage) were produced per sample, of which 81% mapped properly to the *F. vesca* genome. The average non-bisulfite conversion rate among the samples was 0.10 (See Table S1 for more details). To calculate global methylation ratios, output files from wgbs pipeline were pre-filtered for a minimum coverage of 5 reads using awk command and only the common cytosine positions were kept among all the samples using bedtools 2.28.0. The data were tested for statistical significance with an unpaired Student’s t-test. p < 0.05 was selected as the point of minimal statistical significance in all the analyses. R-packages ggplot2 v.3.3.5 and gplots v.3.1.1 were used for the visualization of the results. Global DNA methylation levels were computed by combing all bedGraph files of the individual samples into a unionbedg file and filtered for cytosine positions without sequencing coverage and average DNA methylation levels calculated.

### Identification of differentially methylated regions (DMRs)

First, bedGraph files from wgbs pipeline were pre-filtered for a minimum coverage of 5 reads using awk command. These output files were then used as input for the EpiDiverse/dmr bioinformatics analysis pipeline for non-model plant species to define DMRs [32] with default parameters (minimum coverage threshold 5; maximum q-value 0.05; minimum differential methylation level 10%; 10 as minimum number of Cs; Minimum distance (bp) between Cs that are not to be considered as part of the same DMR is 146 bp). The pipeline uses metilene v.0.2.6.1 (https://www.bioinf.uni-leipzig.de/Software/metilene/) for pairwise comparison between groups and R-packages ggplot2 v.3.3.5 and gplots v.3.1.1, for visualization results (Fig. S1). Based on our *F. vesca* genome transcript annotation and methylation data (overlapped regions with DNA methylation cytosines and DMRs), we detected the methylated genes, promoters, 3’ UTRs, 5’UTR and transposable elements in strawberry. Global DNA methylation and DMR plots were performed with R-package ggplot2. Gene analyses by methylation patterns and analysis of per-family TE DNA methylation profiles were performed with deepTools v.3.5.0. DMRs comparison between treatments were done by the Venn diagram v.1.7.0 R-package. We produced several genome browsers tracks with DMRs that we integrated in our local instance of JBrowse available at the following url: https://jbrowse.agroscope.info/jbrowse/?data=fragaria_sub.

### Heat stress and salt stress RNA-seq analysis

One-month-old seedlings on agarose plates were exposed to heat stress (37°C). For salt stress, plants were transferred to MS media supplemented with 100 mM sodium chloride (NaCl). The stresses were carried out for 2 weeks with 2 recovery days after one week. Following the recovery days, 5 plants were pooled to reduce the variability among individuals (three biological replicates per condition) and flash frozen in liquid nitrogen for transcriptome sequencing. RNA extractions were performed using NucleoSpin RNA Plus, Mini kit for RNA purification with DNA removal column (cat# 740984.50). Samples were sent in heat, salt, and control condition (n = 9) for Illumina paired-end reads sequencing (150 bps) to Novogene (Hongkong, China).

### Differential gene expression analysis

RNA-seq analyses were performed as previously described in [3]. Briefly FastQC v.0.11.9 and trimmomatic v.0.39 packages were used for quality control and trimming. ﻿Salmon v1.4.0 was used to map sequence reads to the *F. vesca* reference transcript dataset. DESeq2 package was use for the quantitative differential gene transcription analysis at the European Galaxy platform with default parameters (Fig. S2).

### Gene ontology (GO) and Kyoto encyclopedia of genes and genomes (KEGG) analysis of differentially methylated and expressed genes

All methylated and differentially expressed genes were annotated based on GO and KEGG annotation downloaded from the Genome Database for Rosaceae (GDR) (https://www.rosaceae.org/species/fragaria_vesca/genome_v4.0.a2).

To better understand the potential function of the differentially methylated and expressed genes, GO functional classification of these genes was performed by AgriGOv1.2 [38]. Genes and promoters were classified by genes contained hypo and hypermethylated DMRs. Similarly, all DEGs were extracted with p-adj < 0.05. The GO slim library was used as reference GO reference type. Fisher’s exact test p-values were calculated for over-representation of the differential methylated genes in all GO categories and Hochberg (FDR) as multi-test adjust method. GO terms with p < 0.05 were considered as significantly enriched. KEGG analysis was performed with the R-package: clusterProfiler [39]; pvalueCutoff = 0.05; pAdjustMethod = “fdr”. Plots were performed with R-package ggplot2.

## Acknowledgements

The European Training Network “EpiDiverse” received funding from the EU Horizon 2020 program under Marie Skłodowska-Curie grant agreement No 764965; The European Research Council (ERC) under the European Union’s Horizon 2020 research and innovation program; No 725701 “BUNGEE” to E.B.. Funding for open access charge: Agroscope institutional funding. This study was supported by INRAE, Angers-Nantes, France and Agroscope, Nyon-Switzerland. We would like to thank all the members of the EpiDiverse consortium (www.epidiverse.eu) and the Crop Genome Dynamics research group for invaluable support, Katharina Jandrasits for preparing WGBS libraries and Dr. Marta Robertson for the careful reading of the manuscript.

## Author contribution

ME.L and E.B conceived the study. ME.L performed the experiments, analyzed sequencing data and wrote the manuscript. D.R. assembled the *F. vesca* genome and wrote the manuscript. C.B. performed experiments and wrote the manuscript. B.D provided plant material and wrote the manuscript. E.B. designed experiments, analyzed data, set up the genome browser and wrote the manuscript.

## Data availability

The datasets generated and/or analyzed in this study are available in the Zenodo repository (DOI: 10.5281/zenodo.6141713). The sequencing data from this study have been submitted to European Nucleotide Archive (ENA, www.ebi.ac.uk/ena/, accessed on ERP135585) under the project PRJEB50996. The Bisulfite-sequencing raw read fastq accessions under ERR8684931-ERR8684954. The raw reads of the RNA-sequencing under the accessions: ERS11547432- ERS11547437 and ERS12247535-ERS12247537.

## Competing interests

The authors declare they have no conflicts of interest.

## Supplementary data

Supplementary data is available at *Horticulture Research * online.

## Supplementary Material

Web_Material_uhac174
