# Supplementary material for: DNA methylation dynamics during stress response in woodland strawberry (Fragaria vesca)
Source: Hortic Res. 2022 Aug 4;9:uhac174. doi: 10.1093/hr/uhac174 (PMC9533225; doi:10.1093/hr/uhac174)
Supplement: Web_Material_uhac174 [file web_material_uhac174.zip › Supplementary_Figures_Tables.pdf]

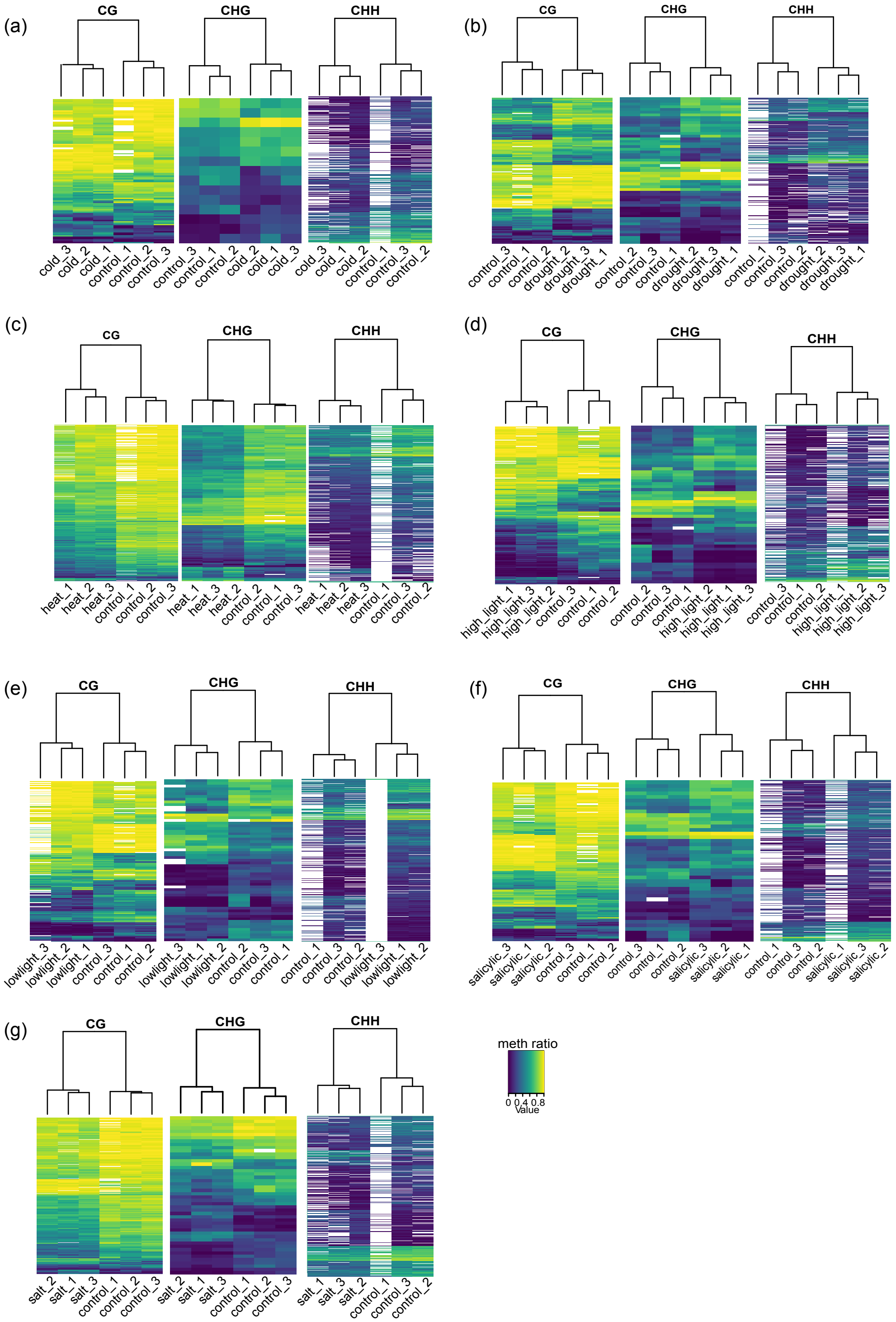

Fig. S1 Heatmaps of significant DMRs (q < 0.05) in CG, CHG and CHH. Methylome comparisons from control plants vs a stress condition: (a) cold, (b) drought, (c) heat, (d) high light, (e) low light, (f) SA, (g) salt.

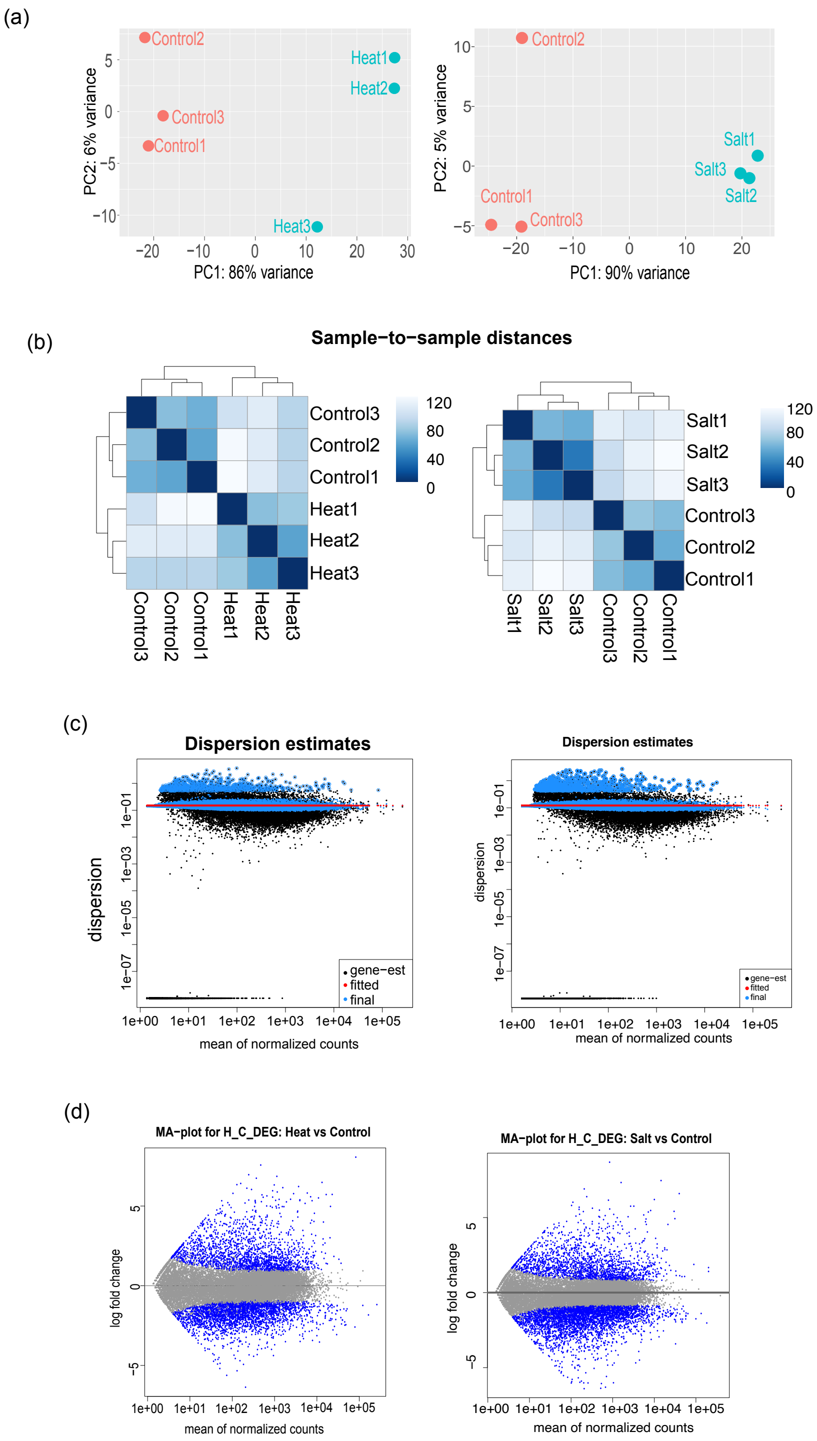

Fig. S2 RNA-seq analysis to detect differentially expressed genes (DEGs). (a) PCA plot showing the variability among control, heat stress and salt stress samples. (b) Heatmap and cluster showing an overview of similarities and differences among samples. (c) Dispersion plot of the mean of the normalized counts. (d) Volcano plot showing the log2 fold change given to the variable mean of normalized counts. Blue points indicates if the adjusted p-value is less than 0.1. Plots were obtained by using DESeq2 in the Galaxy platform.

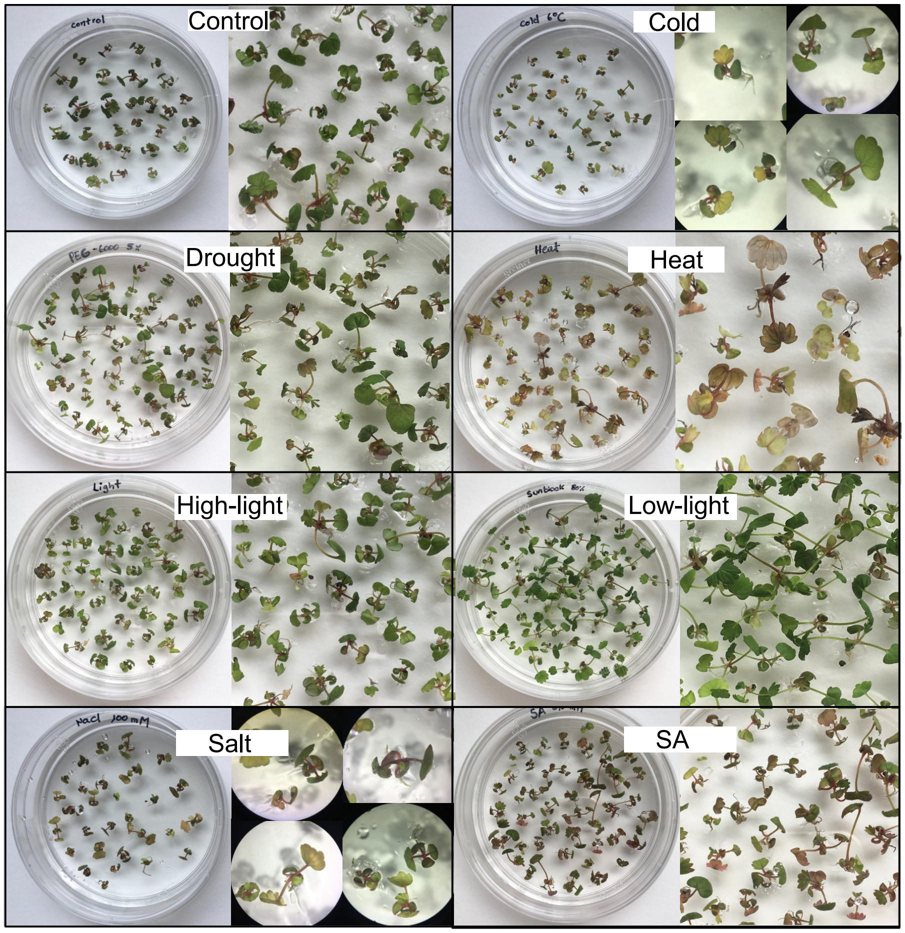

Fig. S3 ***F. vesca*** **development under abiotic and hormonal stress**. Close ups of photographs of plates with one-month-old plants grown under different stress conditions (cold, drought, heat, high- and low intensity of light, salt, and salicylic acid). Plants showed a noticeable reduced size growing under cold stress and salt stress conditions compared to controls. High temperature caused tissue necrosis and reduced chlorophyl content. High intensity of light affected plant development meanwhile low light caused petiole elongation. Smaller roots were observed in drought stress, salt stress, and SA stress. In addition, plants exposed to SA had reddish color suggesting the accumulation of anthocyanins.

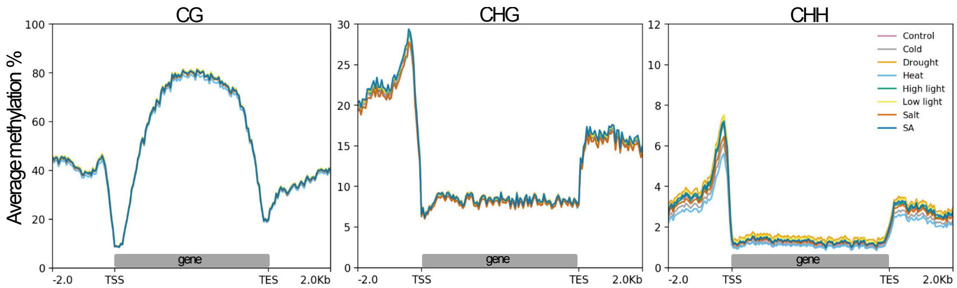

Fig. S4 Global methylation plot profiles of genes with body methylation (gbM). Plots show distribution of DNA methylation in CG, CHG and CHH context around genes classified as genes with body methylation (gbM) with and without stress (Control). Mean of the average methylation percentage (within a sliding 100-bp window) was plotted 2 kb upstream of TSS, over the gene body and 2 kb downstream of TES.

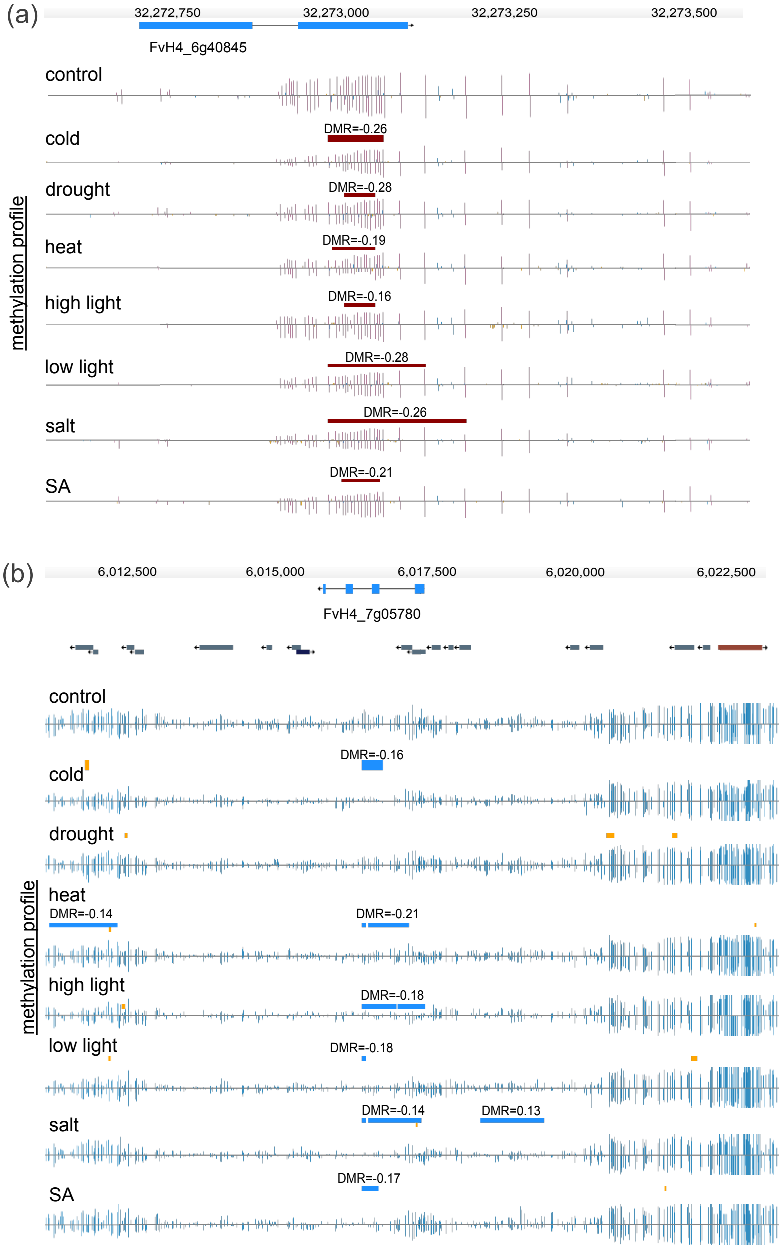

Fig. S5 Common stress induced DMRs per context in promoter and genic regions. (a) Overlapping CG-DMRs with different methylation profiles in all stress conditions. (b) CHG-DMRs diverge in different stress conditions and are enriched in gene bodies. Genome browser view methylation profiles derived from whole genome bisulfite sequencing. Boxes above the histograms indicate identified DMRs (color codes for DNA methylation: red for CG, blue for CHG and yellow for CHH contexts).

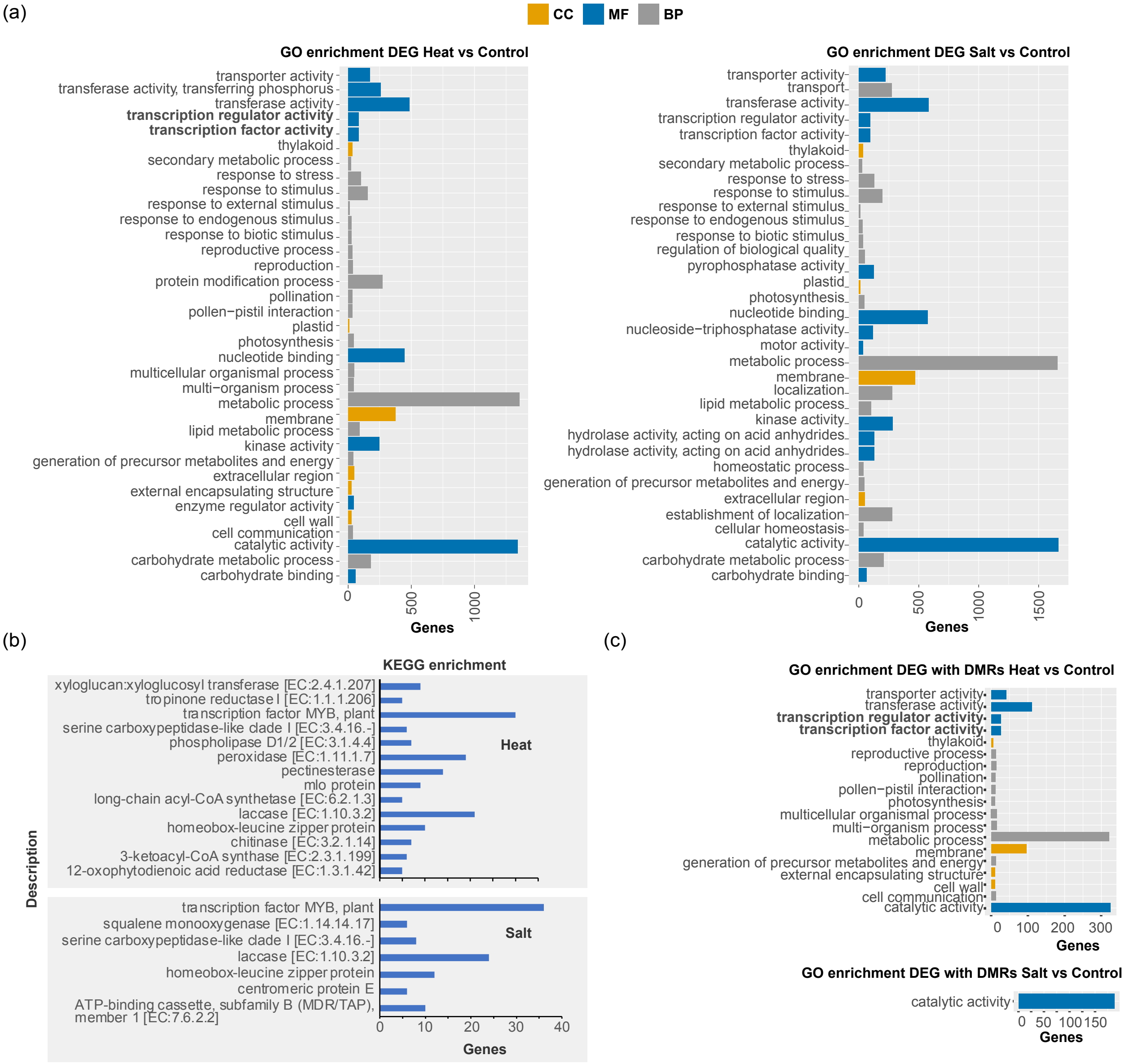

Fig. S6 Functional analysis of differentially expressed genes (DEG) after heat *and salt* stress. (a) Singular enrichment analysis (SEA) of the total number of DEGs after two days of recovery from heat and salt stress (results from AgriGOv2, p-adj:<0.05). (b) KEGG enrichment analysis of the total number of DEGs after two days of recovery from heat and salt stress (results from clusterProfiler, p-adj:<0.05). (c) Singular enrichment analysis (SEA) of DEGs related with DMRs in genic regions (promoter and gene body). The x-axis indicates the number of genes in a category. The y-axis indicates the most enriched GO terms in three categories: biological processes (BP, grey), cellular component (CC, orange), and molecular function (MF, dark blue). The *x*-axis indicates the number of genes in a category.

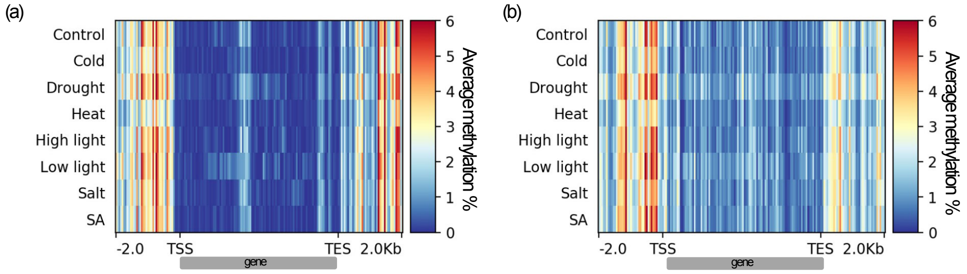

Fig. S7 Global CHH methylation distribution over transcription factors under abiotic and hormone stress conditions. (a) APETALA2/ethylene-responsive element binding protein (*AP2*/*EREBP*) superfamily. (b) Heat shock transcription factors *(HSF)*. Heatmaps showing distribution of DNA methylation in CHH context around genes with and without stress (Control). Mean of the average methylation percentage (within a sliding 100-bp window) was plotted 2 kb upstream of TSS, over the gene body and 2 kb downstream of TES.

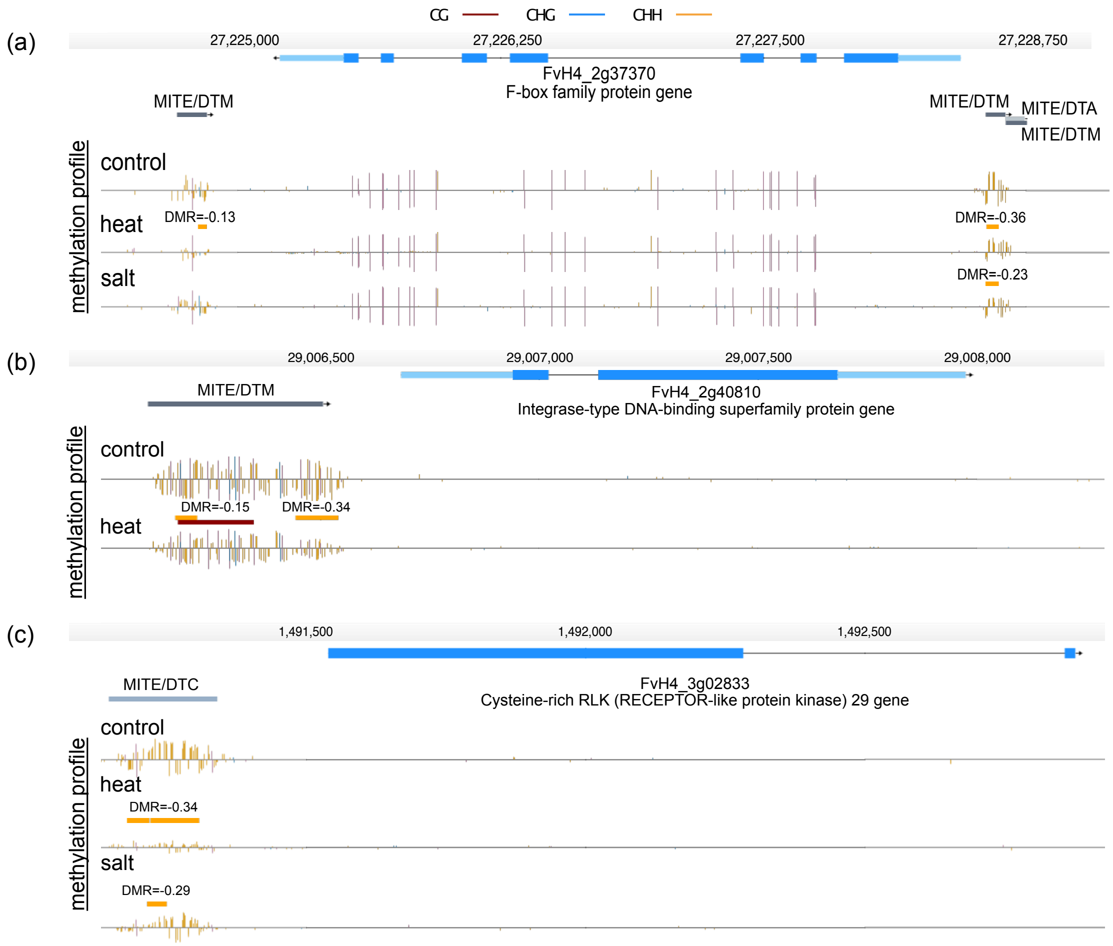

Fig. S8 ***Miniature Inverted-Repeat Transposons*** **(MITEs) present hypomethylated regions (DMRs) under abiotic stress conditions.** Genome browser views of DMRs present in MITEs located near genes. (a) F-box family protein gene (FvH4_2g37370). (b) Integrase-type DNA binding superfamily protein gene (FvH4_2g40810). (c) Cysteine-rich RLK (RECEPTOR-like protein kinase) 29 (FvH4_3g02833). Depicted are genes structures (top panels, UTRs in light blue, exons in blue), TEs (red and dark blue) and DNA methylation levels (histograms). Boxes above the histograms indicate identified DMRs with methylation difference ratio (color codes for DNA methylation: red for CG, blue for CHG and yellow for CHH contexts).

Table S1. Bisulfite sequencing data quality analysis

Sample	Treatment	Tissue	Tech.rep.	strategy	source	selection	layout	platform	Model	Total raw sequences	reads mapped	%	reads mapped and paired	properly paired reads (%)	Non-conversion Rate
FV_FR_01_01_P0_IC0_S1	control	Seedling	1	Bisulfite-Seq	GENOMIC	RANDOM	paired	ILLUMINA	HiSeq X Ten	54350112	43194568	79.47	41103610	89.6	0.0849765
FV_FR_01_01_P0_IC0_S2	control	Seedling	1	Bisulfite-Seq	GENOMIC	RANDOM	paired	ILLUMINA	HiSeq X Ten	75685306	61502655	81.26	59033115	88.7	0.0957948
FV_FR_01_01_P0_IC0_S3	control	Seedling	1	Bisulfite-Seq	GENOMIC	RANDOM	paired	ILLUMINA	HiSeq X Ten	88196726	71859155	81.47	68666898	88.5	0.0824866
FV_FR_01_01_P0_IT0_S1	heat	Seedling	1	Bisulfite-Seq	GENOMIC	RANDOM	paired	ILLUMINA	HiSeq X Ten	82033770	63336642	77.20	60355952	87.1	0.158832
FV_FR_01_01_P0_IT0_S2	heat	Seedling	1	Bisulfite-Seq	GENOMIC	RANDOM	paired	ILLUMINA	HiSeq X Ten	95891176	78466389	81.82	76205355	90.1	0.0658496
FV_FR_01_01_P0_IT0_S3	heat	Seedling	1	Bisulfite-Seq	GENOMIC	RANDOM	paired	ILLUMINA	HiSeq X Ten	131601020	107575452	81.74	104747479	89.6	0.0489135
FV_FR_01_01_P0_IF0_S1	cold	Seedling	1	Bisulfite-Seq	GENOMIC	RANDOM	paired	ILLUMINA	HiSeq X Ten	75356018	62062067	82.35	60460228	89.9	0.0557463
FV_FR_01_01_P0_IF0_S2	cold	Seedling	1	Bisulfite-Seq	GENOMIC	RANDOM	paired	ILLUMINA	HiSeq X Ten	113833012	92894326	81.60	90539656	89.5	0.0523164
FV_FR_01_01_P0_IF0_S3	cold	Seedling	1	Bisulfite-Seq	GENOMIC	RANDOM	paired	ILLUMINA	HiSeq X Ten	62087752	51227464	82.50	49991019	89.3	0.0709958
FV_FR_01_01_P0_IN0_S1	salt	Seedling	1	Bisulfite-Seq	GENOMIC	RANDOM	paired	ILLUMINA	HiSeq X Ten	65068602	54636225	83.96	53200844	91.2	0.113733
FV_FR_01_01_P0_IN0_S2	salt	Seedling	1	Bisulfite-Seq	GENOMIC	RANDOM	paired	ILLUMINA	HiSeq X Ten	100511800	84669826	84.23	82590659	90	0.0612594
FV_FR_01_01_P0_IN0_S3	salt	Seedling	1	Bisulfite-Seq	GENOMIC	RANDOM	paired	ILLUMINA	HiSeq X Ten	80760776	67889432	84.06	66159488	89.7	0.0599842
FV_FR_01_01_P0_IS0_S1	SA	Seedling	1	Bisulfite-Seq	GENOMIC	RANDOM	paired	ILLUMINA	HiSeq X Ten	57969684	47564328	82.05	46310855	89.6	0.0529798
FV_FR_01_01_P0_IS0_S2	SA	Seedling	1	Bisulfite-Seq	GENOMIC	RANDOM	paired	ILLUMINA	HiSeq X Ten	91659264	71433867	77.93	69656974	90.4	0.062181
FV_FR_01_01_P0_IS0_S3	SA	Seedling	1	Bisulfite-Seq	GENOMIC	RANDOM	paired	ILLUMINA	HiSeq X Ten	117736938	96487769	81.95	93542164	89	0.0828464
FV_FR_01_01_P0_IB0_S1	low light	Seedling	1	Bisulfite-Seq	GENOMIC	RANDOM	paired	ILLUMINA	HiSeq X Ten	85964910	71004318	82.59	68857406	90	0.0754448
FV_FR_01_01_P0_IB0_S2	low light	Seedling	1	Bisulfite-Seq	GENOMIC	RANDOM	paired	ILLUMINA	HiSeq X Ten	81716372	66908386	81.87	64790729	89.8	0.0829156
FV_FR_01_01_P0_IB0_S3	low light	Seedling	1	Bisulfite-Seq	GENOMIC	RANDOM	paired	ILLUMINA	HiSeq X Ten	29863420	24419321	81.77	23741172	89.9	0.232153
FV_FR_01_01_P0_ID0_S1	drought	Seedling	1	Bisulfite-Seq	GENOMIC	RANDOM	paired	ILLUMINA	HiSeq X Ten	109809600	91100946	82.96	88787544	89.9	0.109024
FV_FR_01_01_P0_ID0_S2	drought	Seedling	1	Bisulfite-Seq	GENOMIC	RANDOM	paired	ILLUMINA	HiSeq X Ten	94456118	77908248	82.48	75691073	85.3	0.301095
FV_FR_01_01_P0_ID0_S3	drought	Seedling	1	Bisulfite-Seq	GENOMIC	RANDOM	paired	ILLUMINA	HiSeq X Ten	97889484	80948114	82.69	78542173	89.1	0.0902901
FV_FR_01_01_P0_IL0_S1	high light	Seedling	1	Bisulfite-Seq	GENOMIC	RANDOM	paired	ILLUMINA	HiSeq X Ten	54431008	44156034	81.12	42787879	88.7	0.0792454
FV_FR_01_01_P0_IL0_S2	high light	Seedling	1	Bisulfite-Seq	GENOMIC	RANDOM	paired	ILLUMINA	HiSeq X Ten	79622196	64766856	81.34	62804109	89.6	0.0868833
FV_FR_01_01_P0_IL0_S3	high light	Seedling	1	Bisulfite-Seq	GENOMIC	RANDOM	paired	ILLUMINA	HiSeq X Ten	60025776	48357441	80.56	46943047	90.2	0.0792454

Table S2. Summary of Differentially methylated regions (DMRs) in strawberry seedling grown at normal and stress conditions

		Median			Mean				
Treatment	DMR	number_C	met-diff	bps	number_C	met-diff	bps	total DMRS	Context
Cold	hypermethylated	12	0.14	143	15	0.17	182	45	CG
Cold	hypomethylated	12	-0.13	123	14	-0.16	157	59	CG
Drought	hypermethylated	10	0.17	142	13	0.20	162	115	CG
Drought	hypomethylated	12	-0.23	136	10	-0.23	165	83	CG
Heat	hypermethylated	10	0.17	121	12	0.19	140	117	CG
Heat	hypomethylated	12	-0.15	155	13	-0.18	183	2899	CG
Highlight	hypermethylated	12	0.19	136	13	0.22	155	81	CG
Highlight	hypomethylated	12	-0.25	135	14	-0.24	161	99	CG
Lowlight	hypermethylated	12	0.14	129	14	0.18	162	110	CG
Lowlight	hypomethylated	12	-0.18	138	14	-0.20	174	126	CG
SA	hypermethylated	12	0.14	144	15	0.17	163	76	CG
SA	hypomethylated	12	-0.16	122	14	-0.17	161	109	CG
Salt	hypermethylated	12	0.14	131	14	0.17	162	74	CG
Salt	hypomethylated	12	-0.16	143	14	-0.18	180	357	CG
Cold	hypermethylated	15	0.18	279	29	0.19	447	8	CHG
Cold	hypomethylated	26	-0.20	456	31	-0.18	482	7	CHG
Drought	hypermethylated	18	0.19	251	22	0.20	303	32	CHG
Drought	hypomethylated	22	-0.23	243	28	-0.22	411	23	CHG
Heat	hypermethylated	19	0.21	265	24	0.22	335	26	CHG
Heat	hypomethylated	15	-0.23	211	19	-0.23	270	210	CHG
Highlight	hypermethylated	21	0.23	288	23	0.24	342	27	CHG
Highlight	hypomethylated	17	-0.22	256	26	-0.23	406	26	CHG
Lowlight	hypermethylated	18	0.22	179	20	0.22	263	23	CHG
Lowlight	hypomethylated	19	-0.22	212	22	-0.23	300	38	CHG
SA	hypermethylated	22	0.20	282	27	0.19	364	28	CHG
SA	hypomethylated	26	-0.19	303	30	-0.20	381	16	CHG
Salt	hypermethylated	24	0.17	429	25	0.19	388	14	CHG
Salt	hypomethylated	15	-0.17	204	20	-0.19	285	34	CHG
Cold	hypermethylated	11	0.13	40	13	0.14	49	1308	CHH
Cold	hypomethylated	13	-0.16	45	15	-0.16	54	5041	CHH
Drought	hypermethylated	13	0.14	47	15	0.15	58	4475	CHH
Drought	hypomethylated	11	-0.14	41	13	-0.15	48	1158	CHH
Heat	hypermethylated	12	0.13	42	14	0.14	52	1037	CHH
Heat	hypomethylated	13	-0.17	48	16	-0.18	58	11377	CHH
Highlight	hypermethylated	13	0.16	46	15	0.17	57	2960	CHH
Highlight	hypomethylated	12	-0.14	42	14	-0.15	51	1349	CHH
Lowlight	hypermethylated	12	0.16	44	14	0.17	54	4775	CHH
Lowlight	hypomethylated	12	-0.14	42	13	-0.15	50	2210	CHH
SA	hypermethylated	12	0.15	44	15	0.16	53	2802	CHH
SA	hypomethylated	12	-0.15	43	13	-0.16	51	1266	CHH
Salt	hypermethylated	12	0.13	45	14	0.15	53	2064	CHH
Salt	hypomethylated	13	-0.16	45	15	-0.17	54	4350	CHH

Table S3. Percentage of genes used for GO enrichment analysis

Treatment	Genes	Genes with GO number	Genes with no GO number	% Analyzed genes
Cold	2934	1545	1389	52.66
Drought	2471	1267	1204	51.27
Heat	6873	3765	3108	54.78
High light	1804	896	908	49.67
Low light	2985	1553	1432	52.03
Salt	3047	1614	1433	52.97
SA	1820	927	893	50.93

Table S6. APETALA2/ethylene-responsive element binding protein (AP2/EREBP) superfamily which present DMRs in all different contexts.

Gene ID	genome	symbol	treatment	meth_diff	DMR size (bp)	context	Fold change (log2) heat stress	p-adj heat	Fold change (log2) salt stress	p-adj salt
FvH4_1g16350	promoter	*AP2-1*	cold	-0.13881	40	CHH	-1.135029634	0.03392564	-2.157686357	4.63368E-08
			heat	-0.162917	47	CHH				
FvH4_6g34710	promoter	*AP2-15*	high light	0.130002	63	CHH	-1.537082675	0.01473786	-0.711307751	0.455802305
FvH4_7g04950	promoter	*AP2-17*	cold	-0.12849	54	CHH	-0.683737646	0.47495521	-0.427161587	0.828481621
			low light	0.178205	32	CHH				
FvH4_3g33940	promoter	*AP2-5*	cold	-0.237724	29	CHH	0.399505668	0.88318089	0.488969911	0.541070592
FvH4_2g22290	promoter	*ERF12*	heat	-0.235965	164	CG	-1.303119445	0.17230057	0.109516393	0.940634193
FvH4_2g29150	promoter	*ERF14*	heat	-0.128772	70	CHH	-1.553446664	0.02332213	-2.047520196	6.84482E-05
			salt	-0.147	48	CHH				
FvH4_2g40810	promoter	*ERF15*	heat	-0.358406	98	CHH	0.752630674	0.32749548	2.560944499	1.98906E-08
				-0.190833	49	CHH				
				-0.147292	172	CG				
FvH4_3g19450	promoter	*ERF19*	heat	-0.144147	23	CHH	-0.304711524	0.93702748	0.781456898	0.198153961
FvH4_3g23440	promoter	*ERF20*	heat	-0.135556	85	CHH	-0.512605902	0.6713991	-0.157065598	0.940634193
				-0.105729	96	CG				
FvH4_4g03450	promoter	*ERF22*	heat	-0.245888	73	CHH	2.169043248	0.00298674	1.300001295	0.098406254
			low light	0.163993	39	CHH				
FvH4_4g03470	gene	*ERF24*	heat	-0.255333	115	CG	1.602443965	0.15184826	1.494503823	0.016234967
			salt	-0.181481	159	CG				
FvH4_4g10480	promoter	*ERF26*	high light	0.170966	69	CHH	-0.651983242	0.93702748	0.364874836	0.940634193
FvH4_1g23350	promoter	*ERF3*	heat	-0.279487	73	CHH	2.35513607	0.00020395	1.310325545	0.017054355
			low light	0.206927	68	CHH				
FvH4_4g22650	promoter	*ERF30*	heat	-0.416944	137	CG	2.23876655	1.1793E-06	1.756807407	5.60359E-05
			salt	-0.253611	137	CG				
FvH4_4g27491	promoter	*ERF32*	heat	-0.227619	186	CG	-0.017923728	0.98049072	-0.476954557	0.508020759
FvH4_5g03840	promoter	*ERF33*	heat	-0.148553	134	CHH	-0.006035678	0.99334512	-0.866469191	0.083389596
FvH4_5g09520	promoter	*ERF36*	heat	-0.198205	47	CHH	4.987851049	1.3173E-24	3.918540802	1.10187E-16
FvH4_2g06050	promoter	*ERF4*	heat	-0.188491	85	CHH	0.952967143	0.55790632	-0.012586391	0.991744647
			heat	-0.168611	79	CHH				
			SA	0.138918	71	CHH				
FvH4_5g19800	promoter	*ERF40*	salt	-0.187255	82	CHH	-0.563855463	0.66795721	0.235640448	0.940634193
FvH4_5g19840	promoter	*ERF41*	heat	-0.209	26	CHH	-0.359989394	0.92994259	-0.301487533	0.87390843
FvH4_6g28880	promoter	*ERF45*	high light	0.213818	48	CHH	0.075312832	0.94574417	-1.261560376	0.354024351
FvH4_6g29930	promoter	*ERF47*	high light	-0.112958	27	CHH	-2.525570205	9.5301E-05	-2.248393398	8.39248E-05
			salt	-0.148333	39	CHH				
FvH4_6g42000	promoter	*ERF49*	heat	-0.244	49	CHH	-0.128447431	0.93702748	-0.255006022	0.940634193
			heat	-0.122	43	CHH				
			SA	-0.103111	43	CHH				
FvH4_2g06060	promoter	*ERF5*	heat	-0.24881	57	CHH	-0.456570487	0.93702748	-0.836225077	0.820088805
			heat	-0.12029	74	CHH				
FvH4_7g10070	promoter	*ERF53*	salt	0.145064	60	CHH	-2.533771327	0.00148471	-1.604233508	0.019120213
FvH4_7g15860	promoter	*ERF55*	heat	-0.175397	85	CHH	-1.042490271	0.19027345	0.664503867	0.572143777
FvH4_7g26930	promoter	*ERF59*	heat	-0.266349	69	CHH	-1.104241791	0.02781568	0.089769973	0.940634193
FvH4_2g13240	promoter	*ERF6*	heat	-0.279	104	CG	4.674102484	1.8497E-15	3.818729203	6.24209E-20
FvH4_7g26940	promoter	*ERF60*	heat	-0.219706	43	CHH	-1.758068066	0.00030967	-1.718923179	7.82299E-06
			salt	0.175691	47	CHH				
FvH4_7g30930	promoter	*ERF61*	salt	0.147333	22	CHH	1.532457532	0.01321432	1.120631625	0.080626882

FvH4_2g21550	promoter	*ERF7*	heat	-0.29661	23	CHH	0.823966217	0.37353056	-0.007972356	0.992268373
FvH4_5g01440	promoter	*FvDREB1*	heat	-0.252986	216	CHH	0.204476706	0.93702748	-0.341439002	0.940634193
			heat	0.150667	39	CHH				
			salt	-0.205	112	CHH				
FvH4_5g19440	promoter	*FvDREB13*	drought	-0.251	128	CG	-1.607764583	0.0577693	-2.172419333	0.002896007
FvH4_6g18090	promoter	*FvDREB18*	heat	-0.138596	68	CHH	0.129921286	0.93702748	0.094386683	0.940634193
FvH4_7g09550	promoter	*FvDREB20*	heat	-0.134035	90	CHH	-1.277082235	0.04367409	-1.994883059	8.04712E-06
FvH4_1g09180	promoter	*FvDREB23*	heat	-0.165238	60	CHH	-3.321375912	0.00070981	0.51870019	0.940634193
			heat	-0.143	61	CHH				
FvH4_1g16370	promoter	*FvDREB24*	low light	0.108581	34	CHH	-0.537251492	0.63827038	-0.503664891	0.615318517
FvH4_5g33180	promoter	*FvDREB27*	high light	0.209042	36	CHH	1.378213238	0.14634024	2.041320695	0.002404844
FvH4_1g21210	promoter	*FvDREB29*	heat	-0.336667	118	CG	0.414983188	0.90322945	-0.075708698	0.940634193
				-0.262745	90	CHH				
FvH4_5g37820	promoter	*FvDREB31*	heat	-0.370333	83	CG	0.712642917	0.34844381	0.388096018	0.741964998
				-0.17534	88	CG				
				-0.120926	130	CHH				
FvH4_6g26090	promoter	*FvDREB32*	drought	0.237534	27	CHH	2.876719478	5.0649E-05	2.502445079	4.01403E-05
			heat	-0.178805	28	CHH				
			heat	-0.178739	33	CHH				
FvH4_6g43870	promoter	*FvDREB7*	cold	-0.292202	28	CHH	-0.694333512	0.37241636	-0.771468006	0.165298003
			heat	-0.186389	21	CHH				
			heat	-0.179399	30	CHH				
FvH4_3g44200	promoter	*RAV1*	heat	-0.190302	104	CHH	0.129921286	0.93702748	0.094386683	0.940634193
			salt	-0.149703	69	CHH				
FvH4_6g45390	promoter	*RAV7*	heat	-0.150833	76	CHH	-0.102451784	0.94574417	-0.006577338	0.996527964
			heat	-0.142	51	CG				
			high light	-0.19079	43	CHH				

* Transcriptional changes after heat stress are showed in fold change (log2).

Table S7. Heat shock transcription factors (HSFs) which contains DMRs under stress.

Gene ID	genome	symbol	treatment	meth_diff	DMR size (bp)	context	Fold change (log2) heat stress	p-adj	Fold change (log2) salt stress	p-adj
FvH4_5g01770	promoter	FvHsfB2b	heat	-0.2475	116	CG	0.595495148	0.61620172	0.150319766	0.94063419
	promoter		heat	-0.230725	97	CHH				
FvH4_6g22550	-	FvHsfA4b	-	-	-	-	-0.529371975	0.59118496	-0.979810992	0.04280449
FvH4_4g13230	promoter	FvHsfA5a	heat	-0.112075	132	CG	0.421045159	0.79877443	0.325891282	0.81530107
FvH4_3g07170	promoter	FvHsfA4a	cold	-0.184167	43	CHH	-0.021204613	0.9768485	-0.296039189	0.93823701
FvH4_2g23000	promoter	FvHsfA6a	heat	-0.191714	186	CG	-0.665751201	0.51384088	-0.690508833	0.25291289
FvH4_4g01180	promoter	FvHsfA6a	heat	-0.178667	42	CHH	-0.665751201	0.51384088	-0.53619085	0.53779542
FvH4_4g33360	promoter	FvHsfA8a	heat	-0.168614	57	CHH	0.076586732	0.93702748	-0.091049922	0.94063419
FvH4_3g09340	-	FvHsfB2a	-	-	-	-	0.629275143	0.48108199	-0.250915282	0.94063419
FvH4_7g29190	TES	FvHsfB2a	heat	-0.183001	258	CHG	0.629275143	0.48108199	0.473889446	0.59671356
	TES		heat	-0.158826	32	CHH				
FvH4_2g34260	-	FvHsfB4a	-	-	-	-	-1.69373206	0.1247633	-0.824213136	0.62516822
FvH4_1g04810	promoter	FvHsfC1a	heat	-0.16278	46	CHH	-1.111434276	0.10252929	-0.987793019	0.09077002
FvH4_5g22720	promoter	FvHsfA1d	cold	-0.105129	44	CHH	0.482817918	0.75456012	0.136763397	0.94063419
	promoter		heat	-0.109583	66	CHH				
	promoter		heat	-0.105714	43	CHH				
FvH4_1g07800	promoter	FvHsfA9a	drought	0.123981	133	CHH	0.868980506	0.31799828	1.137828001	0.0212134
	promoter		SA	0.23	37	CHH				
FvH4_2g31690	-	FvHsfA2a	-	-	-	-	-0.133955015	0.93702748	0.016437006	0.9826631
FvH4_6g17890	-	FvHsfA3a	-	-	-	-	0.82431526	0.43673661	0.511174988	0.79795151
FvH4_6g24120	promoter	FvHsfB3a	heat	-0.123865	160	CG	2.653069104	1.0933E-08	2.918911485	6.3639E-12
FvH4_3g16090	promoter	FvHsfA1b	cold	-0.147773	167	CHH	0.196107649	0.93702748	-0.117010138	0.94063419
	promoter		heat	-0.300605	41	CHH				
	promoter		salt	-0.13751	154	CHH				
FvH4_1g16030	promoter	FvHsfB1a	salt	0.127333	67	CHH	2.881481593	5.2258E-08	2.92221771	3.7699E-09
	promoter		salt	0.122637	38	CHH				
FvH4_6g27900	promoter	FvHsfA4c	cold	-0.212994	37	CHH	0.43167242	0.78093559	0.302310529	0.88664386
	promoter		cold	-0.231754	34	CHH				
	TES		heat	-0.153333	124	CG				
	promoter		drought	0.326217	167	CHH				
	promoter		low light	0.272328	48	CHH				
	promoter		salt	0.107894	82	CHH				

* Transcriptional changes after heat stress are showed in fold change (log_2).

Table S8. Differentially expressed *MYB* transcription factors which contains DMRs under heat stress and salt stress.

Heat stress				Salt stress			
GeneID	Gene name	Fold Change	DMR location	GeneID	Gene name	Fold Change	DMR location
FvH4_1g08370	*FvMYB3*	-1.2344793	gene body	FvH4_6g22540	*FvMYB1R78*	-1.1771572	gene body
FvH4_1g02690	*FvMYB1*	-1.3817011	none	FvH4_4g19310	*FvMYB46*	-1.8243113	gene body
FvH4_6g52440	*FvMYB101*	-2.1445243	none	FvH4_5g14661	*FvMYB62*	-1.7730274	gene body
FvH4_7g06630	*FvMYB104*	1.04662657	none	FvH4_6g32950	*FvMYB85*	-1.9942558	gene body
FvH4_7g27910	*FvMYB111*	-1.7599274	none	FvH4_1g02690	*FvMYB1*	-1.4794827	none
FvH4_2g01320	*FvMYB13*	-1.4586106	none	FvH4_1g22020	*FvMYB10*	3.85781188	none
FvH4_2g31070	*FvMYB19*	-2.4132241	none	FvH4_7g01020	*FvMYB102*	-2.1154799	none
FvH4_1g04130	*FvMYB1R1*	-1.500496	none	FvH4_1g29710	*FvMYB11*	-1.2213495	none
FvH4_1g27990	*FvMYB1R14*	-1.9278451	none	FvH4_7g27910	*FvMYB111*	-2.3101476	none
FvH4_2g31190	*FvMYB1R26*	-1.572632	none	FvH4_7g34130	*FvMYB113*	-1.6853809	none
FvH4_2g39660	*FvMYB1R28*	-2.9726072	none	FvH4_2g01320	*FvMYB13*	-1.5700315	none
FvH4_4g35980	*FvMYB1R53*	1.23659414	none	FvH4_2g21500	*FvMYB16*	1.18029894	none
FvH4_5g05280	*FvMYB1R57*	-2.2583359	none	FvH4_2g31070	*FvMYB19*	-1.5842218	none
FvH4_5g16170	*FvMYB1R59*	-1.1788365	none	FvH4_1g04130	*FvMYB1R1*	-2.0043582	none
FvH4_1g13110	*FvMYB1R6*	-3.2475735	none	FvH4_1g27990	*FvMYB1R14*	-2.3930389	none
FvH4_6g22540	*FvMYB1R78*	-1.56368	none	FvH4_2g06880	*FvMYB1R18*	-1.1902405	none
FvH4_7g24880	*FvMYB1R95*	-1.6785048	none	FvH4_2g08910	*FvMYB1R20*	-1.8078048	none
FvH4_2g31090	*FvMYB21*	-1.35759	none	FvH4_2g31190	*FvMYB1R26*	-2.0010096	none
FvH4_2g35010	*FvMYB26*	-1.4656122	none	FvH4_2g39660	*FvMYB1R28*	-2.0116702	none
FvH4_2g36950	*FvMYB27*	-3.0888009	none	FvH4_2g40620	*FvMYB1R29*	-1.7933467	none
FvH4_3g28890	*FvMYB36*	-1.8682413	none	FvH4_3g14050	*FvMYB1R37*	-2.6807779	none
FvH4_1g08390	*FvMYB4*	2.8374283	none	FvH4_4g15780	*FvMYB1R45*	-1.5112406	none
FvH4_5g00480	*FvMYB53*	-1.7274669	none	FvH4_4g27990	*FvMYB1R49*	-1.1449103	none
FvH4_5g03100	*FvMYB54*	-1.8110075	none	FvH4_4g31230	*FvMYB1R50*	-1.9599206	none
FvH4_5g06070	*FvMYB57*	-1.8328738	none	FvH4_5g05280	*FvMYB1R57*	-2.1434802	none
FvH4_5g14970	*FvMYB63*	-1.6824899	none	FvH4_5g16170	*FvMYB1R59*	-1.4718684	none
FvH4_5g32460	*FvMYB74*	-1.7812376	none	FvH4_1g13110	*FvMYB1R6*	-4.1770838	none
FvH4_5g39550	*FvMYB77*	-1.9580549	none	FvH4_5g18100	*FvMYB1R61*	-2.0617097	none
FvH4_6g08620	*FvMYB79*	-1.8628805	none	FvH4_6g11000	*FvMYB1R73*	-1.7904775	none
FvH4_6g48630	*FvMYB97*	-1.5420272	none	FvH4_6g26660	*FvMYB1R80*	-1.2276216	none
FvH4_1g29710	*FvMYB11*	-1.1867608	promoter	FvH4_6g43450	*FvMYB1R88*	-1.3329385	none
FvH4_3g14050	*FvMYB1R37*	-1.9329305	promoter	FvH4_7g16170	*FvMYB1R92*	-1.2017148	none
FvH4_3g34960	*FvMYB38*	1.44446396	promoter	FvH4_7g17850	*FvMYB1R93*	-1.0864675	none

FvH4_4g19310	*FvMYB46*	-1.6072118	promoter
FvH4_4g31660	*FvMYB51*	-1.5418446	promoter
FvH4_5g19400	*FvMYB69*	-1.8664388	promoter
FvH4_1g22020	*FvMYB10*	3.33667041	TES
FvH4_2g05210	*FvMYB14*	-2.0867636	TES
FvH4_2g08910	*FvMYB1R20*	-2.6023424	TES
FvH4_2g08980	*FvMYB1R21*	-3.5935553	TES
FvH4_2g40620	*FvMYB1R29*	-2.0066378	TES
FvH4_4g27990	*FvMYB1R49*	-1.4571115	TES
FvH4_5g27240	*FvMYB1R67*	-1.5204322	TES
FvH4_4g31000	*FvMYB49*	-2.6853595	TES
FvH4_5g11930	*FvMYB59*	3.9856437	TES
FvH4_5g27870	*FvMYB72*	2.69992748	TES
FvH4_6g32950	*FvMYB85*	-2.3478795	TES
FvH4_6g49630	*FvMYB98*	2.31781248	TES
FvH4_7g23990	*FvMYB1R94*	-1.6772613	none
FvH4_7g24880	*FvMYB1R95*	-1.7119419	none
FvH4_2g31090	*FvMYB21*	-1.2803661	none
FvH4_2g31100	*FvMYB22*	-2.3861838	none
FvH4_2g36950	*FvMYB27*	-3.9232837	none
FvH4_2g38350	*FvMYB28*	-1.5253328	none
FvH4_3g28890	*FvMYB36*	-2.7457565	none
FvH4_3g38150	*FvMYB39*	-0.9472201	none
FvH4_2g36040	*FvMYB3R1*	-1.3114658	none
FvH4_1g08390	*FvMYB4*	3.02534789	none
FvH4_4g03610	*FvMYB43*	1.26987263	none
FvH4_4g31000	*FvMYB49*	-2.7393467	none
FvH4_4g31660	*FvMYB51*	-1.7354851	none
FvH4_5g00480	*FvMYB53*	-1.9355429	none
FvH4_5g03100	*FvMYB54*	-2.5862492	none
FvH4_5g06070	*FvMYB57*	-1.1591517	none
FvH4_5g11930	*FvMYB59*	2.90495506	none
FvH4_5g14970	*FvMYB63*	-1.7709598	none
FvH4_5g17111	*FvMYB66*	1.79404963	none
FvH4_5g17120	*FvMYB67*	-1.8111976	none
FvH4_5g17970	*FvMYB68*	-1.3671282	none
FvH4_5g19400	*FvMYB69*	-2.4320786	none
FvH4_5g32460	*FvMYB74*	-1.831831	none
FvH4_6g08620	*FvMYB79*	-1.651571	none
FvH4_6g45380	*FvMYB94*	-1.0824522	none
FvH4_6g49630	*FvMYB98*	2.18001706	none
FvH4_4g24870	*FvMYB1R48*	-1.101203	promoter
FvH4_6g15160	*FvMYB1R75*	-1.3759482	promoter
FvH4_5g15900	*FvMYB65*	-1.5467714	promoter
FvH4_6g16480	*FvMYB83*	1.15273926	promoter
FvH4_2g08980	*FvMYB1R21*	-2.6597899	TES

Table S9. Association of stress-induced differentially methylated regions with transposable elements in *F. vesca*

TE	TOTAL counts in genome
DNA/DTA	10867
DNA/DTC	14437
DNA/DTH	6119
DNA/DTM	27122
DNA/DTT	1190
DNA/Helitron	19743
long_terminal_repeat	2434
LTR/Copia	12750
LTR/Gypsy	12690
LTR/unknown	27460
MITE/DTA	2293
MITE/DTC	225
MITE/DTH	2289
MITE/DTM	5898
MITE/DTT	7

(a) Total number of TE in the *Fragaria vesca* genome

TE family	cold	drought	heat	high_light	low_light	SA	salt
DNA/DTA	512	470	974	314	546	347	504
DNA/DTC	500	463	998	429	649	373	510
DNA/DTH	253	242	650	166	299	160	251
DNA/DTM	1215	1158	2366	804	1343	807	1203
DNA/DTT	45	34	121	33	43	22	41
DNA/Helitron	504	427	1294	374	533	309	516
LTR/Copia	421	373	817	307	440	312	445
LTR/Gypsy	546	549	1021	446	690	422	611
LTR/unknown	1313	1323	2464	1007	1535	987	1536

MITE/DTA	143	95	292	92	151	79	142
MITE/DTC	12	5	24	3	6	1	7
MITE/DTH	154	100	327	95	148	65	133
MITE/DTM	391	347	727	206	413	222	370
MITE	700	547	1370	396	718	367	652

(b) Number of TEs with differentially methylated regions.
